# Perturbation Analysis of Quantum Reset Models

**DOI:** 10.1007/s10955-021-02752-y

**Published:** 2021-04-13

**Authors:** Géraldine Haack, Alain Joye

**Affiliations:** 1 Department of Applied Physics, University of Geneva, Chemin de Pinchat 22, 1227 Carouge, Genève Switzerland; 2 Univ. Grenoble Alpes, CNRS, Institut Fourier, 38000 Grenoble, France

**Keywords:** Spectral analysis of Lindbladians; Markovian quantum dynamics; Quantum reset models

## Abstract

This paper is devoted to the analysis of Lindblad operators of Quantum Reset Models, describing the effective dynamics of tri-partite quantum systems subject to stochastic resets. We consider a chain of three independent subsystems, coupled by a Hamiltonian term. The two subsystems at each end of the chain are driven, independently from each other, by a reset Lindbladian, while the center system is driven by a Hamiltonian. Under generic assumptions on the coupling term, we prove the existence of a unique steady state for the perturbed reset Lindbladian, analytic in the coupling constant. We further analyze the large times dynamics of the corresponding CPTP Markov semigroup that describes the approach to the steady state. We illustrate these results with concrete examples corresponding to realistic open quantum systems.

## Introduction

A major challenge when investigating small quantum systems is to assess their dynamics when coupled to several environments that put the system in an out-of-equilibrium situation. To do so, one often resorts to effective master equations governing the reduced density operator for the small system. Under the Born-Markov approximation (that involves weak system-bath coupling and short bath time-correlations), the evolution equation for the reduced density operator becomes linear, and is cast into the form of a Lindblad-type master equation [12, 13] for the corresponding map to be CPTP (Completely Positive and Trace Preserving). A Hamiltonian approach using perturbation theory is probably the most standard way to derive such a (continuous in time) effective evolution equation for the reduced quantum system [5, 28]. For an account of mathematical results, we refer the reader to the review [10] and to the recent paper [22] which implements this procedure rigorously for a general class of systems. Alternatively, repeated-interaction schemes (discrete in time) have attracted lots of attention among both mathematicians [1, 7–9, 14, 18] and physicists [2, 3, 20, 25, 26, 29, 31], especially in the context of quantum thermodynamics. Exact solutions for the asymptotic steady states generated by both types of dynamics can in general be derived for quantum systems with low dimensional Hilbert space only.

Appealing master equations to investigate the dynamics of higher dimensional quantum systems are provided by a specific class of models, known as Quantum Reset Models (QRM hereafter). These models can be viewed as a natural extension of classical stochastic models, see [16] for a review and [11] for an example treating diffusion processes. Remarkably, QRM can be formulated in terms of Lindblad master equations so that they generate CPTP maps. This is achieved by making specific choices of dissipation channels (corresponding to a fully depolarized quantum channel), see [15, 23, 33] for examples in specific physical setups. These QRM, thanks to their structural simplicity, present the strong advantage to allow for analytical solutions for the reduced density operator of multipartite quantum systems and have been successfully exploited to assess the dynamics of specific quantum systems, namely small quantum thermal machines made of a few qubits, qutrits or higher-dimensional quantum systems [4, 6, 19, 30, 32, 33].

In this work, we raise the question to which extent general properties of the dynamics generated by QRM can be analyzed mathematically. Our aim is to go beyond specific models to determine generic properties of the dynamics of QRM, *i.e.* induced by the mathematical structure itself of the QRM. A first step in that direction is performed in the recent work [27] where a single system driven by a Lindbladian subject to a reset process is considered. The spectral properties of the total Lindbladian perturbed by the reset processes are established, under the assumption that the unperturbed Lindbladian possesses a unique stationary state. Extensions to certain degenerate unperturbed Lindbladians are also discussed and examplified. In the present paper, we consider QRM describing the dynamics of more complex structures that are therefore intrinsically degenerate and not amenable to the cases dealt with above. We reach a two-fold objective. On the one hand, we show that those degenerate QRM nevertheless allow for a complete mathematical treatment revealing a rich structure. On the other hand, we demonstrate the relevance of our perturbative analysis to assess the dynamics of realistic multipartite quantum systems characterized by Hilbert spaces of dimension as high as 8.

More precisely, our generic model is made of a tripartite structure, $A-C-B$, where *A* and *B* are the two quantum systems subject to reset processes, and *C* is a central system with its own free evolution. The three subsystems are weakly interacting through a Hamiltonian. We first recall that QRM are always characterised by Lindblad generators, with explicit dissipators. Then we analyse the spectral properties of the resulting Lindbladians and the dynamics of the tri-partite system they generate, under generic hypotheses on the coupling term. We conduct this analysis first in absence of interaction between the $A-C-B$ parts of the Hilbert space they are defined on, which gives rise to an uncoupled Lindbladian displaying large degeneracies, *i.e.* a large subspace of invariant states. Then, we introduce a generic interaction between these different parts and perform a perturbative analysis in the coupling constant. We prove uniqueness of an invariant steady state under the coupled dynamics, analytic in the coupling constant, and provide a description of the converging power series of this non-equilibrium steady state that develops in the small system. Building up on our spectral analysis, we elucidate the long time properties of the dynamics of the tri-partite system and its approach to the steady state. Finally, we focus on the case where the uncoupled system has no Hamiltonian drive and we describe in particular the emergence of a natural classical Markov process in the description of the large time behaviour of the coupled system. The paper closes with the study of two examples illustrating the key features of this analysis: the systems A and B are two qubits while the central system C is of arbitrary dimension *N* and the uncoupled dynamics has no Hamiltonian drive. For a rather general choice of QRM coupled dynamics, we compute the leading order of the steady state for *N* arbitrary and, for $N=2$—when C is another qubit—we determine the steady state up to order three in the coupling constant as well as the associated classical Markov process.

## Mathematical Framework

### Simple Hilbert Space Setup

As a warmup, we consider a single quantum system of finite dimension characterized by its Hamiltonian *H* defined on its Hilbert space $\mathcal{H}$ which is coupled to *M* reservoirs. QRMs assume the state of the quantum system to be reset to a given state $\tau _l$ with probability $\gamma _l \, dt$ within each time interval *dt*. The QRM-type evolution equation is given by [4, 15, 19]:

2.1 $$\begin{aligned} \dot{\rho }(t)=-i [H, \rho ]+\sum _{l=1}^M\gamma _l(\tau _l \, \mathrm{tr}(\rho )-\rho )\,. \end{aligned}$$

The operator $\rho$ is the reduced density operator of the system defined on $\mathcal{H}$, and $\gamma _l$ characterizes the coupling rate to the reservoir *l*, $l= 1, \ldots , M$.

For the sake of comparison with our main concern—tri-partite systems—and to set the notation, we discuss the dynamics of QRM defined in this simple setup, essentially along the lines of [27]. We provide a full description of its generic features, under the following assumptions.

**Gen**:

Let $\mathcal{H}$ be a Hilbert space, with $\dim \mathcal{H}=N<\infty$. The dissipative part of the generator is characterised by

- $\{\tau _l\}_{1\le l \le M}$ a collection of density matrices on $\mathcal{H}$, *i.e.* $\tau _l\in \mathcal{B}(\mathcal{H})$, with $\tau _l\ge 0$ and $\mathrm{tr}(\tau _l)=1$, for all $l\in {1, \dots , M}$,
- $\gamma _l >0$, $l\in {1, \dots , M}$, the collection of associated non-zero rates for the coupling to the *M* baths.

The Hamiltonian part of the generator, $H=H^*\in \mathcal{B}(\mathcal{H})$, is generic in the spectral sense

- $\sigma (H)=\{e_1, e_2, \dots , e_N\}$, consists of simple eigenvalues with associated normalised eigenvectors denoted by $\{\varphi _j\}_{1\le j\le N}$, *i.e.* $H\varphi _j = e_j\varphi _j$, $j\in \{1, \cdots , N\}$,
- The differences (Bohr frequencies) $\{e_j-e_k\}_{j\ne k}$ are all distinct.

The generator of QRM is thus the (super-)operator $\mathcal{L}\in \mathcal{B}(\mathcal{B}(\mathcal{H}))$ defined by

2.2 $$\begin{aligned} \mathcal{L}(\rho )=-i [H, \rho ]+\sum _{l=1}^M\gamma _l(\tau _l \, \mathrm{tr}(\rho )-\rho ), \end{aligned}$$

where $\rho$ here is arbitrary in $\mathcal{B}(\mathcal{H})$, such that the dynamics of the QRM reads

2.3 $$\begin{aligned} \dot{\rho }(t)=\mathcal{L}(\rho (t)), \ \ t\in (0,\infty ), \ \ \rho (0)=\rho _0\in \mathcal{B}(\mathcal{H}). \end{aligned}$$

In case $\rho \in \mathcal{DM(H)}$, the set of density matrices $\mathcal{DM(H)}=\{\rho \in \mathcal{B}(\mathcal{H}) \, | \, \rho \ge 0, \mathrm{tr}(\rho )=1\}$, the trace factor in (2.2) disappears. Indeed, we will see below in a more general framework that the operator $\mathcal{L}$ enjoys further symmetries, being a Lindblad operator, see Proposition 3.2; in particular if $\rho _0\in \mathcal{DM(H)}$, $\rho (t)\in \mathcal{DM(H)}$, for all $t\in [0,\infty )$.

However, we perform the full spectral analysis of $\mathcal{L}$ as an operator on $\mathcal{B}(\mathcal{H})$ and, accordingly, solve the equation (2.3) without resorting to these symmetries.

We first combine the density matrices $\tau _l$ with corresponding rates $\gamma _l$ into a single density matrix *T* with corresponding rate $\Gamma$. Setting

2.4 $$\begin{aligned} \Gamma =\sum _{l=1}^M\gamma _l>0, \ \ T=\frac{1}{\Gamma }{\sum _{l=1}^M} \gamma _l \tau _l\in \mathcal{DM(H)}, \end{aligned}$$

we get that (2.2) writes

2.5 $$\begin{aligned} \mathcal{L}(\rho )=-i[H,\rho ]+\Gamma (T\mathrm{tr}(\rho )-\rho ). \end{aligned}$$

In the sequel, we denote the matrix elements of any $A\in \mathcal{B}(\mathcal{H})$ in the basis $\{\varphi _j\}_{1\le j\le N}$ by $A_{jk}=\langle \varphi _j | A \varphi _k\rangle$, and the operator $|\varphi \rangle \langle \psi |\in \mathcal{B}(\mathcal{H})$, for $\varphi , \psi \in \mathcal{H}$, is defined by $|\varphi \rangle \langle \psi | : \eta \mapsto \varphi \langle \psi |\eta \rangle$.

#### Lemma 2.1

Under our assumptions **Gen**, the operator $\mathcal{L}: \mathcal{B}(\mathcal{H})\rightarrow \mathcal{B}(\mathcal{H})$ defined by (2.5) is diagonalisable with spectrum given by

2.6 $$\begin{aligned} \sigma (\mathcal{L})=\{0, -\Gamma \}\cup \{-i(e_j-e_k)-\Gamma \}_{j\ne k}\}. \end{aligned}$$

All eigenvalues are simple, except $-\Gamma$ which has multiplicity $N-1$.

Moreover, the solution to (2.3) reads

2.7 $$\begin{aligned} \rho (t)=e^{-t(i[H,\cdot ]+\Gamma )}\big (\rho _0-{\mathrm{tr}}(\rho _0)\Gamma \big (i[H,\cdot ]+\Gamma \big )^{-1}(T) \big )+{\mathrm{tr}}(\rho _0)\Gamma \big (i[H,\cdot ]+\Gamma \big )^{-1}(T). \end{aligned}$$

Expressed in the eigenbasis of *H*, this means that, with $\lambda _{jk}=i(e_j-e_k)+\Gamma$,

2.8 $$\begin{aligned} \rho _{jk}(t)=e^{-t\lambda _{jk}} {\rho _0}_{jk}+ {\mathrm{tr}}(\rho _0)\Gamma \frac{T_{jk}}{\lambda _{jk}}\big (1-e^{-t\lambda _{jk}} \big ), \ \ \text{ for } \text{ all } \ 1\le j,k\le N. \end{aligned}$$

#### Remark 2.2

i) In the limit $t\rightarrow \infty$ the steady state is independent of the initial condition and reads
  2.9 $$\begin{aligned} \rho ^{SS} \equiv \lim _{t\rightarrow \infty }\rho (t)=\Gamma \big (i[H,\cdot ]+\Gamma \big )^{-1}(T) \end{aligned}$$
ii) In particular, for $\rho _0\in \mathcal{DM(H)}$, all populations decay to $T_{jj}$ at the same exponential rate without oscillations $\rho _{jj}(t)=e^{-t\Gamma } {\rho _0}_{jj}+ T_{jj}\big (1-e^{-t\Gamma }\big )$.
iii) The result is known, see *e.g.* [27]; we provide a proof for the sake of comparison with those of the sections to come.

#### Proof

We first deal with the dynamical aspects and note that $\mathcal{L}(\cdot ) =-(i[H,\cdot ]+\Gamma \cdot )+\Gamma T\mathrm{tr}(\cdot )$, with $\mathrm{tr}\ T=1$ implies $\mathrm{tr}\mathcal{L}(\rho )=0$ for any $\rho \in \mathcal{B}(\mathcal{H})$, so that the trace is conserved by (2.3). Hence, considering the *jk* matrix element of the differential equation (2.3) we get

2.10 $$\begin{aligned} \dot{\rho }_{jk}=-\lambda _{jk}\rho _{jk}+\Gamma T_{jk}\mathrm{tr}(\rho _0) \ \ \text{ where } \ \ \lambda _{jk}\ne 0, \end{aligned}$$

which yields (2.8). The basis independent formulation (2.7) follows by the decomposition $\rho =\sum _{1\le j,k\le N}\rho _{jk}|\varphi _j\rangle \langle \varphi _k|$ and the observation

2.11 $$\begin{aligned} i[H,|\varphi _j\rangle \langle \varphi _k|]+\Gamma |\varphi _j\rangle \langle \varphi _k|=\lambda _{jk}|\varphi _j\rangle \langle \varphi _k|, \end{aligned}$$

which yields $${\big (i[H,\cdot ]+\Gamma \big )^{-1}(T)}_{jk}= T_{jk}/\lambda _{jk}$$.

On the spectral side, the observation above immediately yields $\mathcal{L}(|\varphi _j\rangle \langle \varphi _k|)=-\lambda _{jk}|\varphi _j\rangle \langle \varphi _k|$ for $j\ne k$, showing $\{-\lambda _{jk}\}_{j\ne k}$ are simple eigenvalues by our genericity assumption. To compute the other nonzero eigenvalues of $\mathcal{L}$, we note that if $\rho$ is an eigenvector of $\mathcal{L}$ associated with an eigenvalue $\lambda$, then $\lambda \mathrm{tr}\rho =0$. Hence $\lambda \ne 0$ implies $\mathrm{tr}\rho =0$. Thus, considering the $N-1$ dimensional subspace of diagonal traceless matrices in the eigenbasis of *H*, $\{\rho = \sum _{1\le j\le N} r_j |\varphi _j\rangle \langle \varphi _j| \ \, | \, \, \sum _{1\le j\le N}r_j=0\}$, and making use of the identity $\mathcal{L}(|\varphi _j\rangle \langle \varphi _j|)= \Gamma (T - |\varphi _j\rangle \langle \varphi _j|)$, for any *j*, we see that it coincides with $\mathrm{Ker}\,(\mathcal{L}+\Gamma {\mathbb {I}})$. Finally, the one-dimensional kernel of $\mathcal{L}$ is spanned by $\Gamma \big (i[H,\cdot ]+\Gamma \big )^{-1}(T)$: the inverse is well defined thanks to (2.11), it has matrix elements $\Gamma T_{jk}/\lambda _{jk}$, and trace one. Thus

2.12 $$\begin{aligned} \mathcal{L}(\Gamma \big (i[H,\cdot ]+\Gamma \big )^{-1}(T))=-\Gamma \big (i[H,\cdot ]+\Gamma \cdot \big )(\big (i[H,\cdot ]+\Gamma \big )^{-1}(T))+\Gamma T =0. \end{aligned}$$

$$\square $$

### Tri-partite Hilbert Spaces

We define here the tri-partite systems whose dynamical properties are studied in this paper.

Consider $\mathcal{H}=\mathcal{H}_A\otimes \mathcal{H}_C\otimes \mathcal{H}_B$, where $\mathcal{H}_\#$ are Hilbert spaces, with dimensions noted $n_\#<\infty$, where $\#\in \{A,B,C\}$. Let $\tau _A\in \mathcal{DM(H}_A)$, $\tau _B\in \mathcal{DM(H}_B)$ be two density matrices on their respective Hilbert space and $\gamma _A, \gamma _B>0$ two positive rates. Consider three Hamiltonians $H_A, H_B, H_C$ on their respective Hilbert space that further satisfy

2.13 $$\begin{aligned}{}[H_A,\tau _A]=0, \ \ \text{ and } \ \ [H_B,\tau _B]=0, \end{aligned}$$

while $H_C$ is arbitrary at this point. In applications, the reset state $\tau _\#$ will typically be defined as a Gibbs state at some inverse temperature $\beta _\#$ associated to $H_\#$; *i.e.* $\tau _\#=e^{-\beta _\#H_\#}/Z_\#$ which satisfies (2.13), where $Z_\#$ is the corresponding partition function. In Sect.3, we perform the analysis of the uncoupled case (system $A-C-B$ is non-interacting), and in Sect.4, we make use of analytic perturbation theory to treat the case where a weak interaction is added to the system $A-C-B$.

## The Non-interacting Tripartite QRM

We define the *uncoupled* QRM by the generator

3.1 $$\begin{aligned} \mathcal{L}(\rho )=&-i[H_A\otimes {\mathbb {I}}_{C}\otimes {\mathbb {I}}_{B}+ {\mathbb {I}}_{A}\otimes H_C\otimes {\mathbb {I}}_{B} + {\mathbb {I}}_{A}\otimes {\mathbb {I}}_{C}\otimes H_B ,\rho ] \nonumber \\&+\gamma _A(\tau _A\otimes \mathrm{tr}_A(\rho )-\rho )+\gamma _B(\mathrm{tr}_B(\rho )\otimes \tau _B-\rho ), \end{aligned}$$

where ${\mathbb {I}}_{\#}$ denotes the identity operator on $\mathcal{H}_\#$ and $\mathrm{tr}_\#$ denotes the operator on the tensor product of Hilbert spaces with indices different from $\#$, obtained by taking the partial trace over $\mathcal{H}_\#$. For later purposes, $\mathrm{tr}_{\#\#'}$ denotes the operator on the Hilbert space with index different from $\#$ and $\#'$ obtained by taking the partial trace over $\mathcal{H}_\#\otimes \mathcal{H}_{\#'}$. For example,

3.2 $$\begin{aligned} \mathrm{tr}_A : \mathcal{B}(\mathcal{H}_A\otimes \mathcal{H}_C\otimes \mathcal{H}_B)\rightarrow \mathcal{B}(\mathcal{H}_C\otimes \mathcal{H}_B),\ \ \mathrm{tr}_{AB} : \mathcal{B}(\mathcal{H}_A\otimes \mathcal{H}_C\otimes \mathcal{H}_B)\rightarrow \mathcal{B}(\mathcal{H}_C) \end{aligned}$$

will be viewed as linear maps. We shall abuse notations and write $H_\#$ for the Hamiltonian both on $\mathcal{H}_\#$ and $\mathcal{H}$, the context making it clear what we mean. Also, we shall denote the non-Hamiltonian part of the generator by

3.3 $$\begin{aligned} \mathcal{D}(\rho )=\gamma _A(\tau _A\otimes \mathrm{tr}_A(\rho )-\rho )+\gamma _B(\mathrm{tr}_B(\rho )\otimes \tau _B-\rho ), \end{aligned}$$

so that $$ \mathcal{L}(\rho )=-i\big [H_A+H_C +H_B , \rho \big ]+\mathcal{D}(\rho ). $$

### Remark 3.1

If $n_B=1$, $\mathcal{H}_B\simeq {\mathbb {C}}$ and the last tensor product is trivial. Hence the QRM reduces to $\mathcal{L}(\rho )=-i\big [H_A+H_C, \rho \big ]+\gamma _A(\tau _A\otimes \mathrm{tr}_A(\rho )-\rho )$ on $\mathcal{H}_A\otimes \mathcal{H}_C$, while keeping $\gamma _B>0$.

Let us start by a structural result saying that the QRM at time *t*, $e^{t\mathcal{L}}(\rho _0)$, with $\rho _0$ a state, is a CPTP map, by recalling that its generator can be cast under the form of a Lindblad operator, see e.g. [4, 15, 19]. More precisely, the non-Hamiltonian part of their generator (3.1) takes the form of a dissipator, *i.e.*

3.4 $$\begin{aligned} \sum _jA_j\rho A_j^*-\frac{1}{2}\{A_j^*A_j,\rho \}=\sum _j \frac{1}{2}\Big \{[A_j\rho ,A_j^*]+[A_j,\rho A_j^*]\Big \}, \ \ \text{ for } \ \ A_j\in \mathcal{B}(\mathcal{H}). \end{aligned}$$

Given (3.1), it is enough to consider $\tau _A\otimes \mathrm{tr}_A(\rho )-\rho$ defined on $\mathcal{H}=\mathcal{H}_A\otimes \mathcal{H}_C$.

### Proposition 3.2

Let $\tau _A=\sum _{k}t_k|\varphi _k\rangle \langle \varphi _k|$ be the spectral decomposition of $\tau _A$, where $\{\varphi _k\}_{k}$ is a complete orthonormal basis of $\mathcal{H}_A$. Then

3.5 $$\begin{aligned} \tau _A\otimes {\mathrm{tr}}_A(\rho )-\rho =\sum _{j,k}\big (A_{jk}\rho A_{jk}^*-\frac{1}{2}\{A_{jk}^*A_{jk},\rho \}\big ), \ \ \text{ where } \ \ A_{jk}=\sqrt{t_j}|\varphi _j\rangle \langle \varphi _k|\otimes {\mathbb {I}}_C. \end{aligned}$$

### Remark 3.3

i) This result applies to the non-Hamiltonian part of the generator of QRM defined on a simple Hilbert space as well, by considering $\mathcal{H}_C={\mathbb {C}}$, in which case ${\mathrm{tr}}_A$ reduces to the scalar valued trace.
ii) The operators $A_{jk}$ can be replaced by $\sqrt{t_j}|\varphi _j\rangle \langle \psi _k|\otimes {\mathbb {I}}_C$, where $\{\psi _k\}_k$ is any orthonormal basis of $\mathcal{H}_A$ without altering the result.

### Spectrum of the Uncoupled QRM

We proceed by analysing the spectrum of the uncoupled QRM $\mathcal{L}$ (3.1) in the tri-partite case, making use of the fact that, by construction, the Hamiltonian part of the decoupled QRM commutes with the dissipator as we quickly check:

3.6 $$\begin{aligned}{}[H_A,\cdot ] \circ (\tau _A\otimes \mathrm{tr}_A(\cdot ))(\rho )=[H_A, \tau _A\otimes \mathrm{tr}_A(\rho )]=[H_A,\tau _A]\otimes \mathrm{tr}_A(\rho )=0, \end{aligned}$$

since $\tau _A$ and $H_A$ commute, while

3.7 $$\begin{aligned} (\tau _A\otimes \mathrm{tr}_A(\cdot ))\circ [H_A,\cdot ] (\rho )=\tau _A\otimes (\mathrm{tr}_A (H_A\rho )-\mathrm{tr}_A(\rho H_A))=0, \end{aligned}$$

using $\mathrm{tr}_A(\cdot )=\sum _{j}\langle \varphi _j^A|\otimes {\mathbb {I}}\ \cdot \ |\varphi _j^A\rangle \otimes {\mathbb {I}}$ with $\{\varphi _j^A\}_{1\le j\le n_A}$ an orthonormal basis of eigenvectors of $H_A$. Now, replacing $H_A$ by $H_B$ (or $H_C$ for that matter) yields

3.8 $$\begin{aligned}&[H_B,\cdot ] \circ (\tau _A\otimes \mathrm{tr}_A(\cdot ))(\rho )=\tau _A\otimes [H_B, \mathrm{tr}_A(\rho )], \ \text{ and } \nonumber \\&(\tau _A\otimes \mathrm{tr}_A(\cdot ))\circ [H_B,\cdot ] (\rho )=\tau _A\otimes (\mathrm{tr}_A (H_B\rho )-\mathrm{tr}_A(\rho H_B))=\tau _A\otimes [H_B,\mathrm{tr}_A(\rho )], \end{aligned}$$

since $H_B$ commutes with $\langle \varphi _j^A|\otimes {\mathbb {I}}$ and $|\varphi _j^A\rangle \otimes {\mathbb {I}}$. Altogether, the dissipator and the Hamiltonian parts of $\mathcal{L}$ admit a basis of common eigenvectors that we now determine.

Let us start with the dissipator and its spectral properties.

#### Proposition 3.4

The dissipator, as an operator on $\mathcal{B}(\mathcal{H})$, admits the following spectral decomposition

$$\begin{aligned}&\sigma (\gamma _A(\tau _A\otimes {\mathrm{tr}}_A(\cdot )-{\mathbb {I}})+\gamma _B({\mathrm{tr}}_B(\cdot )\otimes \tau _B-{\mathbb {I}}))=\{0, -\gamma _A, -\gamma _B, -(\gamma _A+\gamma _B)\}\\&\gamma _A(\tau _A\otimes {\mathrm{tr}}_A(\cdot )-{\mathbb {I}})+\gamma _B({\mathrm{tr}}_B(\cdot )\otimes \tau _B-{\mathbb {I}})=0 Q_0-\gamma _A Q_A-\gamma _B Q_B-(\gamma _A+\gamma _B)Q_{AB}, \end{aligned}$$

where the spectral projectors $Q_{\#}$, $\#\in \{0, A, B, AB\}$ are given by

$$\begin{aligned}&Q_0(\rho )=\tau _A\otimes {\mathrm{tr}}_{AB}(\rho )\otimes \tau _B, \\&Q_{AB}(\rho )=\rho -\mathrm{tr}_B(\rho )\otimes \tau _B-\tau _A\otimes \mathrm{tr}_A(\rho )+\tau _A\otimes \mathrm{tr}_{AB}(\rho )\otimes \tau _B, \\&Q_A(\rho )=\big ( \mathrm{tr}_B(\rho )-\tau _A\otimes \mathrm{tr}_{AB}(\rho ) \big )\otimes \tau _B, \ \ Q_B(\rho )=\tau _A\otimes \big ( \mathrm{tr}_A(\rho )- \mathrm{tr}_{AB}(\rho )\otimes \tau _B \big ). \end{aligned}$$

Moreover, the different spectral subspaces in $\mathcal{B}(\mathcal{H})$ are

#### Remark 3.5

i) In case $\gamma _A=\gamma _B$, there are only three distinct eigenvalues and the corresponding spectral projector is $Q_A+Q_B$.
ii) Being spectral projectors, the $Q_{\#}$’s, $\#\in \{0, A, B, AB\}$ satisfy $Q_{0}+Q_{A}+Q_{B}+Q_{AB}={\mathbb {I}}$ and $Q_{\#}Q_{\#'}=\delta _{\#,\#'}Q_\#$
iii) The dimensions referred to correspond to complex dimensions for $\mathcal{B}(\mathcal{H})$.
iv) The result essentially follows from the observation that $\tau _A\otimes {\mathrm{tr}}_A(\cdot )$ and ${\mathrm{tr}}_B(\cdot )\otimes \tau _B$ are commuting projectors.

#### Proof

We start with point iv) of Remark 3.5. For any $\rho$ in $\mathcal{B}(\mathcal{H})$,

3.9 $$\begin{aligned} (\tau _A\otimes {\mathrm{tr}}_A(\cdot ) \circ {\mathrm{tr}}_B(\cdot )\otimes \tau _B)(\rho )&=\tau _A\otimes \mathrm{tr}_{AB}(\rho )\otimes \tau _B=({\mathrm{tr}}_B(\cdot )\otimes \tau _B\circ \tau _A\otimes {\mathrm{tr}}_A(\cdot ))(\rho ) \end{aligned}$$

while $\tau _A\otimes \mathrm{tr}_A(\cdot )\circ \tau _A\otimes {\mathrm{tr}}_A(\cdot )=\tau _A\otimes {\mathrm{tr}}_A(\cdot )$, and similarly for ${\mathrm{tr}}_B(\cdot )\otimes \tau _B$. Hence the dissipator is a linear combination of two commuting projectors to which we can apply the next Lemma. $\square$

#### Lemma 3.6

Let $P, Q \in \mathcal{B}(\mathcal{H})$ such that $P^2=P, Q^2=Q,$ and $[P,Q]=0$. Then, for any $\alpha , \beta \in {\mathbb {C}}$, the identity

3.10 $$\begin{aligned} \alpha P+\beta Q=0({\mathbb {I}}-P)({\mathbb {I}}-Q)+\alpha P({\mathbb {I}}-Q)+\beta Q({\mathbb {I}}-P)+(\alpha +\beta )PQ, \end{aligned}$$

provides the spectral decomposition of $\alpha P+\beta Q$, so that $\sigma (\alpha P+\beta Q)=\{0,\alpha , \beta , \alpha +\beta \}$, with respective spectral projectors $({\mathbb {I}}-P)({\mathbb {I}}-Q), P({\mathbb {I}}-Q), Q({\mathbb {I}}-P), PQ$, and no eigennilpotent.

The proof of the Lemma is immediate, and in case some eigenvalues coincide, the corresponding spectral projector is simply the sum of the individual projectors.

The identifications $P= {\mathbb {I}}-\tau _A\otimes {\mathrm{tr}}_A(\cdot )$, $Q={\mathbb {I}}-{\mathrm{tr}}_B(\cdot )\otimes \tau _B$, $\alpha =-\gamma _A$, $\beta =-\gamma _B$ yield the announced spectral decomposition of the dissipator, together with the explicit spectral projectors. A direct verification then gives the corresponding spectral subspaces. $\square$

The eigenvectors of the Hamiltonian part of $\mathcal{L}$ are readily computed. For $\#\in \{A,B,C\}$, let $\{\varphi _j^\#\}_{1\le j\le n_\#}$ be an orthonormal basis of $\mathcal{H}_\#$ of eigenvectors of $H_\#$, with associated eigenvalues $e^\#_j$, $1\le j\le n_\#$. The eigenvalues need not to be distinct at that point. We denote by $P^\#_{j,k}\in \mathcal{B}(\mathcal{H}_\#)$, $j,k\in \{1,\cdots , n_\#\}$, the operators $P^\#_{j,k}=|\varphi _j^\#\rangle \langle \varphi _k^\#|$ that yield a basis of eigenvectors of the Hamiltonian part of (3.1) of $\mathcal{B}(\mathcal{H}))$:

3.11 $$\begin{aligned}&-i\big [H_A+H_C +H_B , P^A_{j,k}\otimes P^C_{j',k'}\otimes P^B_{j'',k''} \big ]\nonumber \\&\quad =-i(e^A_j-e^A_k+e^C_{j'}-e^C_{k'}+e^B_{j''}-e^B_{k''})P^A_{j,k}\otimes P^C_{j',k'}\otimes P^B_{j'',k''}. \end{aligned}$$

It remains to take into account the role of the trace in the spectral subspaces of the dissipator to get the sought for common basis of eigenvectors of (3.3). To do so, we introduce the $n_\#-1$ dimensional basis of diagonal (w.r.t. to the eigenbasis of $H_\#$) traceless matrices

3.12 $$\begin{aligned} \Delta _j^\#=|\varphi _j^\#\rangle \langle \varphi _j^\#|-|\varphi _{j+1}^\#\rangle \langle \varphi _{j+1}^\#|, \ \ \ j=1, 2, \dots , n_\#-1, \end{aligned}$$

such that $[H_{\#}, \Delta _j^\#]=0$. Together with $\tau _\#$, the $\Delta ^\#_j$’s form a basis of diagonal matrices. Proposition 3.4 then provides the full spectral analysis of the uncoupled QRM .

#### Proposition 3.7

The vectors listed below form a basis of $\mathcal{B}(\mathcal{H})$ consisting in eigenvectors associated with the mentioned eigenvalue of the uncoupled QRM

$\mathcal{L}(\cdot )=-i\big [H_A+H_C +H_B , \cdot \big ]+\mathcal{D}(\cdot )$ defined on $\mathcal{H}=\mathcal{H}_A\otimes \mathcal{H}_C\otimes \mathcal{H}_B$ by (3.1):

$$\begin{aligned}&\tau _A\otimes P^C_{j',k'}\otimes \tau _B\,\leftrightarrow \,-i(e^C_{j'}-e^C_{k'}), \nonumber \\& 1\le j', k' \le n_C\nonumber \\&\Delta _j^A\otimes P^C_{j',k'}\otimes \tau _B \,\leftrightarrow \, -\gamma _A-i(e^C_{j'}-e^C_{k'}), \nonumber \\& 1\le j\le n_A-1, \ 1\le j', k' \le n_C\nonumber \\&P^A_{j,k}\otimes P^C_{j',k'}\otimes \tau _B\,\leftrightarrow \, -\gamma _A-i(e^A_j-e^A_k+e^C_{j'}-e^C_{k'}), \nonumber \\& 1\le j \ne k \le n_A, \ 1\le j', k' \le n_C\nonumber \\&\tau _A\otimes P^C_{j',k'}\otimes \Delta _{j''}^B\,\leftrightarrow \, -\gamma _B-i(e^C_{j'}-e^C_{k'}), \nonumber \\& 1\le j''\le n_B-1, \ 1\le j', k' \le n_C\nonumber \\&\tau _A\otimes P^C_{j',k'}\otimes P^B_{j'',k''}\,\leftrightarrow \, -\gamma _B-i(e^C_{j'}-e^C_{k'}+e^B_{j''}-e^B_{k''}), \nonumber \\& 1\le j'' \ne k'' \le n_B, \ 1\le j', k' \le n_C\nonumber \\&\Delta ^A_{j}\otimes P^C_{j',k'}\otimes \Delta ^B_{j''}\,\leftrightarrow \, -(\gamma _A+\gamma _B)-i(e^C_{j'}-e^C_{k'}), \nonumber \\& 1\le j \le n_A-1, \ 1\le j'' \le n_B-1, \ 1\le j', k' \le n_C\nonumber \\&\Delta ^A_{j}\otimes P^C_{j',k'}\otimes P^B_{j'',k''}\,\leftrightarrow \, -(\gamma _A+\gamma _B)-i(e^C_{j'}-e^C_{k'}+e^B_{j''}-e^B_{k''}), \nonumber \\& 1\le j \le n_A-1, \ 1\le j'' \ne k'' \le n_B, \ 1\le j', k' \le n_C\nonumber \\&{ P^A_{j,k}\otimes P^C_{j',k'}\otimes \Delta ^B_{j''}\,\leftrightarrow \, -(\gamma _A+\gamma _B)-i(e^A_j-e^A_k+e^C_{j'}-e^C_{k'}), } \nonumber \\& 1\le j \ne k \le n_A, \ 1\le j'' \ne n_B-1, \ 1\le j', k' \le n_C\nonumber \\&P^A_{j,k}\otimes P^C_{j',k'}\otimes P^B_{j'',k''}\,\leftrightarrow \, -(\gamma _A+\gamma _B)-i(e^A_j-e^A_k+e^C_{j'}-e^C_{k'}+e^B_{j''}-e^B_{k''}), \nonumber \\& 1\le j \ne k \le n_A, \ 1\le j'' \ne k'' \le n_B, \ 1\le j', k' \le n_C\nonumber \end{aligned}$$

#### Remark 3.8

0) The Hamiltonians $\mathcal{H}_\#\in \mathcal{B}(\mathcal{H}_\#)$ are arbitrary at that point.
i) The uncoupled reset model Lindbladian $\mathcal{L}$ is thus diagonalisable, with eigenvalues located on the (generically) four vertical lines $\mathfrak {R}z=0$, $\mathfrak {R}z=-\gamma _A$, $\mathfrak {R}z=-\gamma _B$, $\mathfrak {R}z=-(\gamma _A+\gamma _B)$ in the complex plane, symmetrically with respect to the real axis.
ii) In particular, the kernel of $\mathcal{L}$ is degenerate, since $\,\dim \mathrm{Ker}\,\mathcal{L}(\cdot )\ge n_C$.
iii) It is straightforward to generalise this result to the case where the dissipator admits a reset part acting on $\mathcal{H}_C$ as well, and to the case of a *p*-partite non interacting system, with $p\in {\mathbb {N}}$ arbitrary.

The spectral projectors of $\mathcal{L}$ can be constructed explicitly, making use of the next Lemma:

#### Lemma 3.9

Consider a Hilbert space $\mathcal{H}$ and $\tau \in \mathcal{B}(\mathcal{H})$ a density matrix. Let $\{\varphi _j\}_{1\le j\le n}$ be an orthonormal basis of eigenvectors of $\tau$ for $\mathcal{H}$. Consider the basis of $\mathcal{B}(\mathcal{H})$ given by

3.13 $$\begin{aligned} P_{jk}=|\varphi _j\rangle \langle \varphi _k|, \ 1\le j\ne k\le n, \ \Delta _j=|\varphi _j\rangle \langle \varphi _j|-|\varphi _{j+1}\rangle \langle \varphi _{j+1}|, \ 1\le j\le n-1, \ \text{ and } \ \tau . \end{aligned}$$

Set $\sigma _j=\sum _{k=1}^{j}|\varphi _k\rangle \langle \varphi _k|$, $1\le j\le n$. Then the operators on $\mathcal{B}(\mathcal{H})$ defined by

3.14 $$\begin{aligned}&Q_{jk}(\cdot )=P_{jk}\mathrm{tr}(P_{jk}^*\, \cdot \, ), \ 1\le j\ne k \le n, \nonumber \\&Q_j(\cdot )=\Delta _j \mathrm{tr}(\sigma _j (\,\cdot -\tau \mathrm{tr}(\cdot ))), \ 1\le j\le n-1, \ \text{ and } \ \ Q_0(\cdot )=\tau \mathrm{tr}({\mathbb {I}}\, \cdot ) \end{aligned}$$

yield a complete set of rank one projectors onto the span of the corresponding basis vectors of (3.13) so that the composition of any two of them equals zero.

#### Remark 3.10

The spectral projectors of $\mathcal{L}$ corresponding to Proposition 3.7 are then given by the appropriate tensor products of projectors (3.14).

The solution to $\dot{\rho }=\mathcal{L}(\rho )$, $\rho (0)=\rho _0$ follows immediately by expanding $\rho _0$ along those eigenvectors. In particular, one gets for this uncoupled QRM model

3.15 $$\begin{aligned} \rho (t)=\tau _A\otimes (e^{-i[H_C,\cdot ]t}\mathrm{tr}_{AB}(\rho _0))\otimes \tau _B+\mathcal{O}(e^{-t\min \{\gamma _A,\gamma _B\}}), \ \ t\ge 0, \end{aligned}$$

where $e^{-i[H_C,\cdot ]t}\mathrm{tr}_{AB}(\rho _0)$ satisfies the Hamiltonian evolution equation $\dot{\rho }_C=-i[H_C,\rho _C]$, $\rho _C(0)=\mathrm{tr}_{AB}(\rho _0)$ on $\mathcal{H}_C$, as expected in this uncoupled context.

## The Weakly-Interacting Tripartite QRM

We consider now the coupled QRM defined by the Lindblad generator on $\mathcal{B}(\mathcal{H})$, with $\mathcal{H}=\mathcal{H}_A\otimes \mathcal{H}_C\otimes \mathcal{H}_B$,

4.1 $$\begin{aligned} \mathcal{L}_g(\rho )=\mathcal{L}(\rho )-ig[H,\rho ]\equiv \mathcal{L}_0(\rho )+g\mathcal{L}_1(\rho ) \end{aligned}$$

where $H=H^* \in \mathcal{B}(\mathcal{H})$ is a Hamiltonian that effectively couples the different Hilbert spaces $\mathcal{H}_\#$, while $g\in {\mathbb {R}}$ is a coupling constant. We focus on the determination of the kernel of $\mathcal{L}_g$, as $g\rightarrow 0$, which describes the asymptotic state of the system driven by $\mathcal{L}_g$, under generic hypotheses. Then we turn to the consequences for the dynamics generated by $\mathcal{L}_g$. By generic hypotheses, we mean that all assumptions we make along the way ensure the coupling is effective enough to lift all degeneracies, so that all accidental degeneracies are eliminated order by order in *g*.

### Leading Order Analytic Perturbation Theory

When $g=0$, Proposition 3.7 shows that

4.2 $$\begin{aligned} \mathrm{Ker}\,\mathcal{L}_0\supset \mathrm{span}\big \{\tau _A\otimes |\varphi _j^C\rangle \langle \varphi _j^C|\otimes \tau _B\big \}_{1\le j\le n_C}, \end{aligned}$$

whatever the properties of the Hamiltonian $H_C$. We shall consider below both cases $H_C=0$ and $H_C\ne 0$, which give rise to different results. In case the Hamiltonian $H_C$ is trivial,

4.3 $$\begin{aligned} H_C=0 \ \Rightarrow \ \mathrm{Ker}\,\mathcal{L}_0= \mathrm{span}\big \{\tau _A\otimes \rho _C\otimes \tau _B\big \}_{\rho _C\in \mathcal{B}(\mathcal{H}_C)} \end{aligned}$$

has dimension $n_C^2$, and the corresponding spectral projector coincides with $Q_0$, the spectral projector on $\mathrm{Ker}\,\mathcal{D}$, see Proposition 3.4. In order to avoid accidental degeneracies when $H_C\ne 0$, we will assume $H_C$ satisfies the spectral hypothesis

**Spec(**$$H_C$$**)**:

The spectrum of $H_C\in \mathcal{B}(\mathcal{H}_C)$ is simple and the Bohr frequencies $\{e^C_j-e_k^C\}_{j\ne k}$ are distinct.

Under this assumption, we have

4.4 $$\begin{aligned} \mathrm{Ker}\,\mathcal{L}_0=\mathrm{span}\big \{\tau _A\otimes \rho _C \otimes \tau _B, \ \text{ s.t. } \ [\rho _C,H_C]=0\big \}, \end{aligned}$$

which is of dimension $n_C$. The corresponding spectral projector acts as follows

4.5 $$\begin{aligned} Q_0(\rho )=\tau _A\otimes \mathrm{Diag}_C(\mathrm{tr}_{AB}(\rho ))\otimes \tau _B, \end{aligned}$$

where the projector $\mathrm{Diag}_C : \mathcal{B}(\mathcal{H}_C)\rightarrow \mathcal{B}(\mathcal{H}_C)$ defined by

4.6 $$\begin{aligned} \mathrm{Diag}_C (\cdot )=\sum _{j=1}^{n_C} | \varphi _j^C\rangle \langle \varphi _j^C| \ \cdot \ | \varphi _j^C\rangle \langle \varphi _j^C| \end{aligned}$$

extracts the diagonal part of $\rho _C$ within the normalised eigenbasis of $H_C$. Observe that $\mathrm{Offdiag}_C : \mathcal{B}(\mathcal{H}_C)\rightarrow \mathcal{B}(\mathcal{H}_C)$, extracting the offdiagonal part of $\rho _C$ within the same basis, yields the complementary projector

4.7 $$\begin{aligned} \mathrm{Offdiag}_C={\mathbb {I}}-\mathrm{Diag}_C. \end{aligned}$$

We also note, for later reference, that $Q_0$ on $\mathcal{B}(\mathcal{H})$ is trace preserving, so that $\mathrm{Ran}\,({\mathbb {I}}-Q_0)\subset \{\rho \, | \, \mathrm{tr}\rho =0\}$.

Analytic perturbation theory, see e.g. Chapter II §2 [17], allows us to compute the splitting of the degenerate eigenvalue zero of $\mathcal{L}_0$ by the perturbation $g\mathcal{L}_1$. Recall here that $\mathcal{L}_g$ being a Lindblad operator (Proposition 3.2), the following structural constraints hold:

4.8 $$\begin{aligned} 0\in \sigma (\mathcal{L}_g)=\overline{\sigma (\mathcal{L}_g)}\subset \{z\in {\mathbb {C}}\ | \mathfrak {R}z\le 0\}, \ \ \forall \ g\in {\mathbb {R}}. \end{aligned}$$

Moreover, the eigenvalue 0 is semisimple, that is there is no eigennilpotent (Jordan block) corresponding to that eigenvalue in the spectral decomposition of $\mathcal{L}_g$. The same is actually true for all eigenvalues sitting on the imaginary axis.

Let $\{\lambda _j(g)\}_{1\le j\le m}$ be the set of eigenvalues of $\mathcal{L}_g$ that stem from the eigenvalue 0 of $\mathcal{L}_0$, with $m=n_C^2$ if $H_C=0$ or $m=n_C$ if $H_C\ne 0$. They form the so-called $\lambda -$group for $\lambda =0$, and for $g\in {\mathbb {C}}\setminus \{0\}$ with |*g*| small enough, $\{\lambda _j(g)\}_{1\le j\le m}$ are analytic functions of a (fractional) power of *g* that tend to zero as $g\rightarrow 0$. These eigenvalues may be permanently degenerate. For the structural reasons recalled above, one of these eigenvalues, denoted by $\lambda _0(g)$, is identically equal to zero, $\lambda _0(g)\equiv 0$, $\forall g\in {\mathbb {C}}\setminus {0}$, and in case $\lambda _0(g)$ is degenerate, it is semisimple.

We show that under generic hypotheses, $\lambda _0(g)\equiv 0$ is a simple eigenvalue, see Theorem 4.3, and we determine the corresponding eigenvector $\rho _0(g)$, normalized to be a state, *i.e.* $\rho _0(g)\ge 0$ and $\mathrm{tr}\rho _0(g)= 1$.

Let us denote by $Q_0(g)$ the analytic spectral projector of $\mathcal{L}_g$ corresponding to the set of eigenvalues in the 0-group . It writes

4.9 $$\begin{aligned} Q_0(g)=\frac{-1}{2i\pi }\int _{\Gamma _0}(\mathcal{L}_g-z)^{-1}dz=Q_0+gQ_1+g^2Q_2+\mathcal{O}(g^3), \end{aligned}$$

for |*g*| small where $\Gamma _0$ is a circle of small radius centered at the origin. Also, since 0 is a semisimple eigenvalue of $\mathcal{L}_0$,

4.10 $$\begin{aligned} Q_1=- Q_0\mathcal{L}_1 S_0-S_0\mathcal{L}_1 Q_0=Q_0Q_1({\mathbb {I}}-Q_0)+({\mathbb {I}}-Q_0)Q_1Q_0, \end{aligned}$$

where $S_0$ is the reduced resolvent of $\mathcal{L}_0$ at 0, satisfying $S_0Q_0=Q_0S_0=0$ and $S_0\mathcal{L}_0=\mathcal{L}_0S_0={\mathbb {I}}-Q_0$. In other words, $S_0=\mathcal{L}_0^{-1}({\mathbb {I}}-Q_0)$, that we shall sometimes abusively write $S_0=\mathcal{L}_0^{-1}$, with the understanding that it acts on $({\mathbb {I}}-Q_0)\mathcal{B}(\mathcal{H})$. The analytic reduced operator in the corresponding subspace which describes the splitting reads

4.11 $$\begin{aligned} Q_0(g)\mathcal{L}_gQ_0(g)&=(Q_0+gQ_1+g^2Q_2+\mathcal{O}(g^3))\nonumber \\&\quad \times (\mathcal{L}_0+g\mathcal{L}_1)(Q_0+gQ_1+g^2Q_2+\mathcal{O}(g^3)) \nonumber \\&= gQ_0 \mathcal{L}_1 Q_0 + g^2 (Q_1 \mathcal{L}_0 Q_1+Q_1\mathcal{L}_1Q_0+ Q_0 \mathcal{L}_1 Q_1)+ \mathcal{O}(g^3), \end{aligned}$$

where we used $\mathcal{L}_0Q_0=Q_0\mathcal{L}_0=0$.

#### Lemma 4.1

Under assumption **Spec(**$H_C$**)** when $H_C\ne 0$, we have

4.12 $$\begin{aligned} Q_0 \mathcal{L}_1 Q_0(\rho )=\left\{ \begin{matrix} 0 &{} \text{ if } \ H_C\ne 0 \\ -i\tau _A\otimes [\overline{H}^{\, \tau }, \mathrm{tr}_{AB}(\rho )]\otimes \tau _B &{} \text{ if } \ H_C= 0 \end{matrix} \right. \end{aligned}$$

where

4.13 $$\begin{aligned} \overline{H}^{\, \tau } :&= \mathrm{tr}_{AB}(\tau _A^{1/2}\otimes {\mathbb {I}}_C \otimes \tau _B^{1/2}\, H\, \tau _A^{1/2}\otimes {\mathbb {I}}_C \otimes \tau _B^{1/2})\nonumber \\&=\mathrm{tr}_{AB}(H\, \tau _A\otimes {\mathbb {I}}_C \otimes \tau _B)=\mathrm{tr}_{AB}(\tau _A\otimes {\mathbb {I}}_C \otimes \tau _B \,H)\in \mathcal{B}(\mathcal{H}_C). \end{aligned}$$

Explicitly, with $$\tau _\#=\sum _{1\le j\le n_\#}t_j^\# | \varphi _j^\#\rangle \langle \varphi _j^\#|$$,

4.14 $$\begin{aligned} \overline{H}^{\, \tau }={\mathop {\mathop {\sum }\limits _{1\le j\le n_A}}\limits _{1\le k\le n_B}}t_j^At_k^B (\langle \varphi _j^A | \otimes {\mathbb {I}}_C\otimes \langle \varphi _k^B|) \ H \ (| \varphi _j^A \rangle \otimes {\mathbb {I}}_C \otimes |\varphi _k^B\rangle ) . \end{aligned}$$

As a consequence, when $H_C\ne 0$ the splitting is generically described by the order $g^2$ correction, while in case $H_C=0$, the non-zero first order correction imposes that the elements of the kernel of $Q_0(g)$ commute with $\overline{H}^{\, \tau }$ which, generically, decreases the degeneracy from $n_C^2$ to $n_C$. In both cases, the eigenvalue zero of $Q_0 \mathcal{L}_1 Q_0$ is semisimple.

#### Proof

We first compute for any $\rho _C\in \mathcal{B}(\mathcal{H}_C)$, using (4.13),

4.15 $$\begin{aligned} \mathrm{tr}_{AB}([H,\tau _A\otimes \rho _C\otimes \tau _B])=[\overline{H}^{\, \tau }, \rho _C]. \end{aligned}$$

One gets the explicit expression for $\overline{H}^{\, \tau }$ by expressing the partial trace within the eigenbases of $\tau _\#$. Therefore

4.16 $$\begin{aligned} Q_0 \mathcal{L}_1 Q_0(\rho )=-i\tau _A\otimes (\mathrm{tr}_{AB}([H,\tau _A\otimes \mathrm{tr}_{AB}(\rho )\otimes \tau _B]))\otimes \tau _B= -i\tau _A\otimes [\overline{H}^{\, \tau }, \mathrm{tr}_{AB}(\rho )]\otimes \tau _B. \end{aligned}$$

The fact that $H_C\ne 0$ implies $Q_0 \mathcal{L}_1 Q_0=0$ then follows from

4.17 $$\begin{aligned} Q_0 \mathcal{L}_1 Q_0(\rho )=-i\tau _A\otimes \mathrm{Diag}_C(\mathrm{tr}_{AB}([H,\tau _A\otimes \mathrm{Diag}_C(\mathrm{tr}_{AB}(\rho ))\otimes \tau _B]))\otimes \tau _B, \end{aligned}$$

and the identity

4.18 $$\begin{aligned} \mathrm{Diag}_C (\mathrm{tr}_{AB}([H,\tau _A\otimes \mathrm{Diag}_C(\rho _C)\otimes \tau _B]))=\mathrm{Diag}_C [\overline{H}^{\, \tau }, \mathrm{Diag}_C (\rho _C)]=0. \end{aligned}$$

$$\square $$

Let us investigate the next order correction in order to analyse the splitting from the eigenvalue zero. Following [17] we consider the analytic matrix

4.19 $$\begin{aligned} \widetilde{\mathcal{L}}_g&=\frac{1}{g}Q_0(g) \mathcal{L}_g Q_0(g) \nonumber \\&= Q_0 \mathcal{L}_1 Q_0 + g (Q_1 \mathcal{L}_0 Q_1+Q_1\mathcal{L}_1Q_0+ Q_0 \mathcal{L}_1 Q_1)+ \mathcal{O}(g^2) \nonumber \\&\equiv \widetilde{\mathcal{L}}_0+g \widetilde{\mathcal{L}}_1 + \mathcal{O}(g^2), \end{aligned}$$

where we observe with (4.10) that

4.20 $$\begin{aligned} \widetilde{\mathcal{L}}_1=- Q_0 \mathcal{L}_1S_0\mathcal{L}_1 Q_0 - S_0\mathcal{L}_1 Q_0 \mathcal{L}_1 Q_0-Q_0\mathcal{L}_1 Q_0 \mathcal{L}_1 S_0. \end{aligned}$$

Let $\widetilde{Q}_0$ be the eigenprojector onto $\mathrm{Ker}\,\widetilde{\mathcal{L}}_0$. Then the spectrum of $\widetilde{Q}_0 \widetilde{\mathcal{L}}_1\widetilde{Q}_0$ describes the splitting to order $g^2$, see [17], Thm 5.11: for $\tilde{\lambda }_j^{(1)}\in \sigma (\widetilde{Q}_0 \widetilde{\mathcal{L}}_1\widetilde{Q}_0)$ of multiplicity $m_j^{(1)}$, there exist exactly $m_j^{(1)}$ eigenvalue of $\mathcal{L}_g$ of the form

4.21 $$\begin{aligned} \lambda _j(g)=g^2\tilde{\lambda }_j^{(1)}+ \mathcal{O}(g^3). \end{aligned}$$

Notice that $\widetilde{Q}_0 \widetilde{\mathcal{L}}_1\widetilde{Q}_0$ is viewed as an operator on $Q_0\mathcal{B}(\mathcal{H})$ here.

We observe that $\widetilde{Q}_0=\widetilde{Q}_0 Q_0 =Q_0 \widetilde{Q}_0$, hence

4.22 $$\begin{aligned} \widetilde{Q}_0 \widetilde{\mathcal{L}}_1\widetilde{Q}_0=-\widetilde{Q}_0 ( Q_0\mathcal{L}_1S_0\mathcal{L}_1 Q_0 )\widetilde{Q}_0=-\widetilde{Q}_0 \mathcal{L}_1\mathcal{L}_0^{-1}\mathcal{L}_1 \widetilde{Q}_0, \end{aligned}$$

since $$\mathcal{L}_1 \widetilde{Q}_0=({\mathbb {I}}-Q_0)\mathcal{L}_1 \widetilde{Q}_0$$.

In order to proceed, we shall also assume in the sequel that the operator $\overline{H}^{\, \tau }$ appearing in Lemma 4.1 has generic spectral properties.

**Spec(**$$\overline{H}^{\, \tau }$$**)**:

The spectrum of $\overline{H}^{\, \tau }\in \mathcal{B}(\mathcal{H}_C)$ is simple and the corresponding Bohr frequencies are distinct. We denote the normalised eigenvectors and eigenvalues of $\overline{H}^{\, \tau }$ by $\varphi _j^\tau$ and $e_j^\tau$, $1\le j\le n_C$.

Under **Spec(**$\overline{H}^{\, \tau }$**)**, we get from (4.5) and Lemma 4.1

4.23 $$\begin{aligned} \widetilde{Q}_0(\rho )=\left\{ \begin{matrix} \tau _A\otimes \mathrm{Diag}_C \mathrm{tr}_{AB}(\rho ) \otimes \tau _B &{} \ \text{ if } \, H_C\ne 0 \\ \tau _A\otimes \mathrm{Diag}_\tau \mathrm{tr}_{AB}(\rho ) \otimes \tau _B &{} \ \text{ if } \, H_C= 0, \end{matrix}\right. \end{aligned}$$

where $\mathrm{Diag}_\tau$ is the projector that extracts the diagonal part of the matrices expressed in the orthonormal eigenbasis $\{\varphi _j^\tau \}$. Therefore

4.24 $$\begin{aligned} \widetilde{Q}_0 \widetilde{\mathcal{L}}_1\widetilde{Q}_0=\tau _A\otimes \mathrm{Diag}\, \mathrm{tr}_{AB}\Big ( \big [H, \mathcal{L}_0^{-1}([H, \tau _A\otimes \mathrm{Diag}\, \mathrm{tr}_{AB}(\cdot ) \otimes \tau _B])\big ]\Big ) \otimes \tau _B, \end{aligned}$$

where $\mathrm{Diag}$ stands here for $\mathrm{Diag}_C$ (resp. $\mathrm{Diag}_\tau$) if $H_C\ne 0$ (resp. $H_C=0$). Equivalently, $\widetilde{Q}_0 \widetilde{\mathcal{L}}_1\widetilde{Q}_0$ is fully characterised by the following linear map. Set

4.25 $$\begin{aligned} \Phi (\cdot ):= {\mathrm{tr}}_{AB}\big (\big [H, \mathcal{L}_0^{-1}([H, \tau _A\otimes \mathrm{Diag}(\, \cdot \,) \otimes \tau _B])\big ]\big ) : \mathcal{B}(\mathcal{H}_C)\rightarrow \mathcal{B}(\mathcal{H}_C)\cap \{\rho _C\, | \, \mathrm{tr}\rho _C=~0\}. \end{aligned}$$

Note that $\Phi$ is well defined and takes the form $\Phi (\rho )=\mathrm{tr}_{AB} ([H, M(\rho )])$, for $M(\rho )\in \mathcal{B}(\mathcal{H})$, hence $\mathrm{tr}\, \Phi (\rho )=\mathrm{tr}([H,M(\rho )])=0$, for any $\rho$. Then, the restriction of $\Phi$ to $\mathrm{Diag}\, \mathcal{B}(\mathcal{H}_C)$, which has dimension $n_C$, satisfies

4.26 $$\begin{aligned} \Phi _D:=\mathrm{Diag}\, \Phi \, |_{\mathrm{Diag}\, \mathcal{B}(\mathcal{H}_C)} \ \ \text{ and } \ \ \widetilde{Q}_0 \widetilde{\mathcal{L}}_1\widetilde{Q}_0(\cdot )=\tau _A\otimes \Phi _D(\cdot )\otimes \tau _B\circ \mathrm{tr}_{AB}(\cdot ). \end{aligned}$$

We shall abuse notations in the sequel and simply write

4.27 $$\begin{aligned} \widetilde{Q}_0 \widetilde{\mathcal{L}}_1\widetilde{Q}_0(\cdot )=\tau _A\otimes \Phi _D(\cdot )\otimes \tau _B, \end{aligned}$$

identifying operators defined on $\widetilde{Q}_0\mathcal{B}(\mathcal{H})$ and $\mathrm{Diag}\, \mathcal{B}(\mathcal{H}_C)$. Hence

4.28 $$\begin{aligned} \sigma (\widetilde{Q}_0 \widetilde{\mathcal{L}}_1\widetilde{Q}_0)=\sigma (\Phi _D). \end{aligned}$$

Note that $\dim \mathrm{Ker}\,\, \Phi _D\ge 1$, since $\mathrm{Ran}\,\, \Phi _D\subset \mathrm{Diag}\, \mathcal{B}(\mathcal{H}_C)\cap \{\rho _C\, | \, \mathrm{tr}\rho _C=~0\}$, a subspace of dimension $n_C-1$, in keeping with the fact that $\mathrm{Ker}\,\mathcal{L}_g$ is never trivial. Hence, for the zero eigenvalue of $\mathcal{L}_g$ to be non-degenerate at second order perturbation in *g*, we assume the coupling satisfies the assumption.

**Coup**:

The linear map

4.29 $$\begin{aligned} \Phi _D (\cdot )=\mathrm{Diag}\, {\mathrm{tr}}_{AB}\big (\big [H, \mathcal{L}_0^{-1}([H, \tau _A\otimes \mathrm{Diag}\, (\, \cdot \,) \otimes \tau _B])\big ]\big ) \ \ \text{ defined } \text{ on } \ \mathrm{Diag}\, \mathcal{B}(\mathcal{H}_C), \end{aligned}$$

where $\mathrm{Diag}$ stands here $\mathrm{Diag}_C$ (resp. $\mathrm{Diag}_\tau$) if $H_C\ne 0$ (resp. $H_C=0$), is such that $\dim \mathrm{Ker}\,\, \Phi _D=1$.

#### Remark 4.2

Assumption **Coup** is equivalent to the statement

$\Phi _D^{-1}$ exists on the $n_c-1$ dimension subspace $\mathrm{Diag}\, \mathcal{B}(\mathcal{H}_C)\cap \{\rho _C\, | \, \mathrm{tr}\rho _C=~0\}=~\mathrm{Ran}\,\Phi _D$.

Indeed, both statements entail $\dim \mathrm{Ker}\,\Phi _D=1$, and the corresponding spectral projector onto $\mathrm{Ker}\,\Phi _D$, say $\Pi _0$, is such that $\mathrm{Ran}\,\Phi _D=({\mathbb {I}}- \Pi _0)\mathrm{Ran}\,\,\Phi _D$, and $\mathrm{Ker}\,\Phi _D \cap \mathrm{Ran}\,\Phi _D=\{ 0\}$.

As a consequence,

#### Theorem 4.3

Consider the coupled QRM $\mathcal{L}_g(\rho )$ defined on $\mathcal{B}(\mathcal{H}_A\otimes \mathcal{H}_C\otimes \mathcal{H}_B)$ by

4.30 $$\begin{aligned} \mathcal{L}_g(\rho )=&-i\big [H_A+H_C +H_B +gH, \rho \big ]+\gamma _A(\tau _A\otimes \mathrm{tr}_A(\rho )-\rho )+\gamma _B(\mathrm{tr}_B(\rho )\otimes \tau _B-\rho ) \end{aligned}$$

and assume **Spec(**$H_C$**)** if $H_C\ne 0$ or **Spec(**$\overline{H}^{\, \tau }$**)** if $H_C=0$. Then for $g\in {\mathbb {C}}\setminus {\{0\}}$, |*g*| small enough, $\dim \mathrm{Ker}\,\, \mathcal{L}_g=1$ if **Coup** holds.

#### Remark 4.4

Under assumption **Spec(**$\overline{H}^{\, \tau }$**)**, the non-zero eigenvalues of $\widetilde{\mathcal{L}}_0$ are all simple, of the form $\lambda _{jk}=-i(e_j^\tau -e_k^\tau )$ with associated eigenvector $\tau _A\otimes |\varphi _j^\tau \rangle \langle \varphi _k^\tau |\otimes \tau _B$, $j\ne k$, and corresponding eigenprojector

4.31 $$\begin{aligned} \widetilde{Q}_{\lambda _{jk}}(\rho )=\tau _A\otimes |\varphi _j^\tau \rangle \langle \varphi _k^\tau |\otimes \tau _B \ \mathrm{tr}(\tau _A\otimes |\varphi _k^\tau \rangle \langle \varphi _j^\tau |\otimes \tau _B \, \rho ). \end{aligned}$$

The next order correction, given by the eigenvalue of the operator $-\widetilde{Q}_{\lambda _{jk}} \mathcal{L}_1\mathcal{L}_0^{-1}\mathcal{L}_1\widetilde{Q}_{\lambda _{jk}}$, reads

4.32 $$\begin{aligned} \tilde{\lambda }_{jk}^{(1)}=\mathrm{tr}\Big \{({\mathbb {I}}_A\otimes |\varphi _k^\tau \rangle \langle \varphi _j^\tau |\otimes {\mathbb {I}}_B)\big [H,\mathcal{L}_0^{-1}([H,\tau _A\otimes |\varphi _j^\tau \rangle \langle \varphi _k^\tau |\otimes \tau _B])\big ]\Big \}. \end{aligned}$$

### Dynamics

We push here the spectral analysis a bit further in order to get sufficient information to analyse the behaviour of the dynamics of the coupled QRM $\mathcal{L}_g(\cdot )$, as $g\rightarrow 0$. We first discuss the richer case $H_C=0$ and then describe the modifications required for the case $H_C\ne 0$.

Let $Q_0(g)$ be the spectral projector of $\mathcal{L}_g$ given by (4.9), and $Q_0^\flat (g)={\mathbb {I}}- Q_0(g)$. We have accordingly

4.33 $$\begin{aligned} e^{t\mathcal{L}_g}=e^{t\mathcal{L}_g^{0}}Q_0(g)+e^{t\mathcal{L}_g^{\flat }}Q_0^\flat (g), \end{aligned}$$

where $\mathcal{L}_g^0=\mathcal{L}_g|_{\mathrm{Ran}\,Q_0(g)}$, and $\mathcal{L}_g^{\flat }=\mathcal{L}_g|_{\mathrm{Ran}\,} Q_0^\flat (g)$. Since the spectrum of $\mathcal{L}_g^{\flat }$ is a positive distance away from the imaginary axis, uniformly in *g* small enough, functional calculus yields the existence of $\Gamma >0$, independent of *g*, such that

4.34 $$\begin{aligned} e^{t\mathcal{L}_g}=e^{t\mathcal{L}_g^{0}}Q_0(g)+\mathcal{O}(e^{-t\Gamma }), \end{aligned}$$

where $\mathcal{O}$ is uniform in *g*, since $Q_0(g)$ is analytic in *g*. Now, by (4.19)

4.35 $$\begin{aligned} \mathcal{L}_g^0=g\widetilde{\mathcal{L}}_g=g(\widetilde{\mathcal{L}}_0+g\widetilde{\mathcal{L}}_1+\mathcal{O}(g^2)), \end{aligned}$$

where, for $H_C=0$ under assumption **Spec(**$\overline{H}^{\, \tau }$**)**,

4.36 $$\begin{aligned} \widetilde{\mathcal{L}}_0=0 \,\widetilde{Q}_0 + \sum _{j\ne k} \lambda _{jk}\widetilde{Q}_{\lambda _{jk}}, \ \text{ where } \ \lambda _{jk}=-i(e^\tau _j-e_k^\tau ), \end{aligned}$$

with simple non zero eigenvalues, see Remark 4.4. In case $H_C\ne 0$ under hypothesis **Spec(**$H_C$**)**, $\widetilde{\mathcal{L}}_0=0$ by Lemma 4.1, so that (4.36) holds with $\widetilde{Q}_0= Q_0$ and $\widetilde{Q}_{\lambda _{jk}}=0$.

Since $\mathcal{L}_g^0=\mathcal{O}(g)$ (and even $\mathcal{L}_g^0=\mathcal{O}(g^2)$ in case $H_C\ne 0$), the long time behaviour of $e^{t\mathcal{L}_g}$ is controlled by the first term in (4.34) when *g* is small. This requires addressing the behaviour of the non self-adjoint spectral projectors associated to eigenvalues of $\mathcal{L}_g$ that vanish as *g* goes to zero.

#### Proposition 4.5

Assuming $H_C=0$, **Spec(**$\overline{H}^{\, \tau }$**)** and **Coup**, there exists $g_0>0$ such that for all $|g|<g_0$, $\mathcal{L}_g$ admits analytic spectral projector $\widetilde{Q}_0(g)$ and $\widetilde{Q}_{\lambda _{jk}}(g)$ and analytic simple eigenvalues $\lambda _{jk}(g)$ such that

4.37 $$\begin{aligned} \mathcal{L}_g^0= g\widetilde{Q}_0(g)\widetilde{\mathcal{L}}_g\widetilde{Q}_0(g) +\sum _{j\ne k}\lambda _{jk}(g)\widetilde{Q}_{\lambda _{jk}}(g). \end{aligned}$$

Here $\widetilde{Q}_0(g) =\widetilde{Q}_0+ \mathcal{O}(g)$, $\widetilde{Q}_{\lambda _{jk}}(g)=\widetilde{Q}_{\lambda _{jk}}+\mathcal{O}(g)$ and $\lambda _{jk}(g)=-ig(e^\tau _j-e_k^\tau )+g^2\tilde{\lambda }_{jk}^{(1)}+\mathcal{O}(g^3)$, see (4.32).

Assuming $H_C\ne 0$, **Spec(**$H_C$**)** and **Coup**, the same statement holds with $\widetilde{Q}_{0}(g)=Q_0+\mathcal{O}(g)$ and $\widetilde{Q}_{\lambda _{jk}}(g)\equiv 0$, $\lambda _{jk}(g)\equiv 0$ in (4.37).

Moreover, assuming **Coup** and **Spec(**$\overline{H}^{\, \tau }$**)**, (respectively **Spec(**$H_C$**)**), if $H_C=0$, (respectively $H_C\ne 0$), we have $\dim \mathrm{Ker}\,\widetilde{Q}_0(g)\widetilde{\mathcal{L}}_g\widetilde{Q}_0(g)\equiv 1$ and the corresponding spectral projector $\widetilde{Q}_0^S(g)$ is analytic for $|g|<g_0$, and satisfies

4.38 $$\begin{aligned} \widetilde{Q}_0^S(g)\mathcal{L}_g=\mathcal{L}_g \widetilde{Q}_0^S(g)=0. \end{aligned}$$

Here

4.39 $$\begin{aligned} \widetilde{Q}_0(g)\widetilde{\mathcal{L}}_g\widetilde{Q}_0(g)=g\widetilde{Q}_0\widetilde{\mathcal{L}}_1\widetilde{Q}_0+\mathcal{O}(g^2)= g \tau _A\otimes \Phi _D \otimes \tau _B+\mathcal{O}(g^2) \end{aligned}$$

and $\widetilde{Q}_0^S(g)=\widetilde{Q}_0^S+\mathcal{O}(g)$ where $\widetilde{Q}_0^S$ is the projector on $\mathrm{Ker}\,\widetilde{Q}_0\widetilde{\mathcal{L}}_1\widetilde{Q}_0$.

#### Remark 4.6

The spectral constraints on Lindblad operators imply,

4.40 $$\begin{aligned} \mathfrak {R}\, \sigma (\widetilde{Q}_0\widetilde{\mathcal{L}}_1\widetilde{Q}_0)\setminus {\{0\}} \le 0, \ \text{ and } \ \mathfrak {R}\tilde{\lambda }_{jk}^{(1)} \le 0. \end{aligned}$$

We give conditions ensuring $\mathfrak {R}\tilde{\lambda }_{jk}^{(1)}<0$ in case the model has no leading order Hamiltonian drive, $\mathcal{L}_0=\mathcal{D}$, that we analyse in more details in Sect. 6.

#### Proof

We consider $H_C=0$ only, the other case being similar. Thanks to (4.35) and (4.36), perturbation theory applies to $\widetilde{\mathcal{L}}_g$ and yields the analytic projectors $\widetilde{Q}_0(g)$ and $\widetilde{Q}_{\lambda _{jk}}(g)$ converging to $\widetilde{Q}_0$ and $\widetilde{Q}_{\lambda _{jk}}$ respectively, and the analytic simple eigenvalues $\lambda _{jk}(g)$, such that (4.37) holds. Expanding the first term using $\widetilde{Q}_0\widetilde{\mathcal{L}}_0= \widetilde{\mathcal{L}}_0\widetilde{Q}_0=0$, one gets thanks to (4.26)

4.41 $$\begin{aligned} \widetilde{Q}_0(g)\widetilde{\mathcal{L}}_g\widetilde{Q}_0(g)= g \widetilde{Q}_0\widetilde{\mathcal{L}}_1\widetilde{Q}_0+\mathcal{O}(g^2)=g\, \tau _A\otimes \Phi _D \otimes \tau _B+\mathcal{O}(g^2). \end{aligned}$$

Assumption **Coup** implies that $\widetilde{Q}_0\widetilde{\mathcal{L}}_1\widetilde{Q}_0$ has one dimensional kernel, with associated spectral projector we write $\widetilde{Q}_0^S$. Hence, perturbation theory again ensures the existence of an analytic one dimensional spectral projector $\widetilde{Q}_0^S(g)$ of $\widetilde{Q}_0(g)\widetilde{\mathcal{L}}_g\widetilde{Q}_0(g)$ corresponding to the simple zero eigenvalue of $\widetilde{Q}_0\widetilde{\mathcal{L}}_1\widetilde{Q}_0$ at $g=0$. Necessarily, $\widetilde{Q}_0^S(g)$ coincides with the spectral projector onto the nontrivial kernel of $\mathcal{L}_g$ for all *g* small enough, which proves (4.38). $\square$

Let us turn to the dynamical implications.

#### Corollary 4.7

Under the hypotheses for $H_C=0$ above, the following holds for all $t\ge 0$ and *g* real small enough:

4.42 $$\begin{aligned} e^{t\mathcal{L}_g}= e^{tg^2(\widetilde{Q}_0\widetilde{\mathcal{L}}_1\widetilde{Q}_0+\mathcal{O}(g))}\widetilde{Q}_0(g)+ \sum _{j\ne k} e^{t\lambda _{jk}(g)}\widetilde{Q}_{\lambda _{jk}}(g)+\mathcal{O}(e^{-t\Gamma }). \end{aligned}$$

Further assuming $\max _{j \ne k} \{\mathfrak {R}\tilde{\lambda }_{jk}^{(1)}\} <0$, there exists $\delta >0$ such that for all $t\ge 0$,

4.43 $$\begin{aligned} e^{t\mathcal{L}_g}&=\widetilde{Q}_0^S(g)+\mathcal{O}(e^{-\delta g^2 t}), \end{aligned}$$

where the constant in the $\mathcal{O}$ is uniform in $t\ge 0$ and *g* small.

Setting $\eta = \min _{j \ne k} \{|\mathfrak {R}\tilde{\lambda }_{jk}^{(1)}|\}>0$, we have

4.44 $$\begin{aligned} e^{t\mathcal{L}_g}&=e^{tg^2\widetilde{Q}_0\widetilde{\mathcal{L}}_1\widetilde{Q}_0}\widetilde{Q}_0+ \mathcal{O}(e^{-tg^2\eta }) + \mathcal{O}(g)+\mathcal{O}(g^3 t)\nonumber \\&=\tau _A\otimes e^{tg^2\Phi _D}\mathrm{Diag}_\tau \mathrm{tr}_{AB} \otimes {\tau _B} + \mathcal{O}(e^{-tg^2\eta }) + \mathcal{O}(g)+\mathcal{O}(g^3 t) \end{aligned}$$

where the constants in all $\mathcal{O}$ are uniform in $t\ge 0$, *g* small.

Under the hypotheses for $H_C\ne 0$ above, for all $t\ge 0$ and *g* real small enough,

4.45 $$\begin{aligned} e^{t\mathcal{L}_g}= e^{tg^2(Q_0\widetilde{\mathcal{L}}_1 Q_0+\mathcal{O}(g))}\widetilde{Q}_0(g)+\mathcal{O}(e^{-t\Gamma }), \end{aligned}$$

and there exists $\delta >0$ such that for all $t\ge 0$,

4.46 $$\begin{aligned} e^{t\mathcal{L}_g}&=\widetilde{Q}_0^S(g)+\mathcal{O}(e^{-\delta g^2 t}), \end{aligned}$$

where the constant in the $\mathcal{O}$ is uniform in $t\ge 0$ and *g* small. Moreover,

4.47 $$\begin{aligned} e^{t\mathcal{L}_g}&=e^{tg^2 Q_0\widetilde{\mathcal{L}}_1 Q_0} Q_0+ \mathcal{O}(e^{-t\Gamma }) + \mathcal{O}(g)+\mathcal{O}(g^3 t)\nonumber \\&=\tau _A\otimes e^{tg^2\Phi _D}\mathrm{Diag}_C \mathrm{tr}_{AB} \otimes {\tau _B} + \mathcal{O}(e^{-t\Gamma }) + \mathcal{O}(g)+\mathcal{O}(g^3 t) \end{aligned}$$

where the constants in all $\mathcal{O}$ are uniform in $t\ge 0$, *g* small.

#### Remark 4.8

0) The identical statements (4.43) and (4.46) show that $1/g^2$ is the time scale of the approach to the asymptotic state, as expected.
i) The full evolution can be approximated by the restriction of $e^{tg^2\tau _A\otimes \Phi _D \otimes \tau _B}$ to $\mathrm{Ran}\,\widetilde{Q}_0$, (provided $\eta$ is larger than the absolute value of the real part of the eigenvalues of $\tau _A\otimes \Phi _D \otimes \tau _B$ in case $H_C=0$).
ii) In case $\mathcal{L}_0=\mathcal{D}$, we provide in Sect. 6 an interpretation of the approximate evolution $e^{tg^2\tau _A\otimes \Phi _D \otimes \tau _B}$ as a classical continuous time Markov process.
iii) Set $F =\max \{|\mathfrak {R}\lambda | \ \lambda \in \sigma (\Phi _D)\}$. When $H_C=0$, the explicit term in (4.44) is the leading term if $F<\eta$, and for times which satisfy $0 \le t < \frac{1}{\epsilon + F}|\ln (g)|/g^2$, as $g\rightarrow 0$, for any $\epsilon >0$. When $H_C\ne 0$, the same is true for the explicit term in (4.47), without constraint on *F*.
iv) This corollary is relevant for an analysis along the lines of [21].

#### Proof

Again we prove the statements for $H_C=0$ only, the other case being similar. The first two statements follow from functional calcul, and Proposition 4.5, taking into account the analyticity of the spectral data involved. To get the last statement, we observe that since the CPTP map $e^{t\mathcal{L}_g}$ has a norm which is uniformly bounded in $t\ge 0$ and *g* small enough, the norm of

4.48 $$\begin{aligned} e^{tg^2(\widetilde{Q}_0\widetilde{\mathcal{L}}_1\widetilde{Q}_0+\mathcal{O}(g))}\widetilde{Q}_0(g)=e^{t\mathcal{L}_g}-\sum _{j\ne k} e^{t\lambda _{jk}(g)}\widetilde{Q}_{\lambda _{jk}}(g)+\mathcal{O}(e^{-t\Gamma }) \end{aligned}$$

is bounded above by a constant $C>0$ which uniform in $t\ge 0$ and *g* small enough. Thus, by Duhamel formula

4.49 $$\begin{aligned} e^{\tau (A+B)}=e^{\tau A}+\int _0^\tau e^{\tau '(A+B)}Be^{(\tau -\tau ')A}d\tau ' \end{aligned}$$

applied to $A=\widetilde{Q}_0\widetilde{\mathcal{L}}_1\widetilde{Q}_0$ subject to (4.40), $B=\mathcal{O}(g)$, $\tau = g^2t$ we get

4.50 $$\begin{aligned} e^{tg^2(\widetilde{Q}_0\widetilde{\mathcal{L}}_1\widetilde{Q}_0+\mathcal{O}(g))}\widetilde{Q}_0(g)= e^{tg^2\widetilde{Q}_0\widetilde{\mathcal{L}}_1\widetilde{Q}_0}\widetilde{Q}_0(g)+\mathcal{O}(g^3 t). \end{aligned}$$

Moreover, $\eta = \min _{j \ne k} \{|\mathfrak {R}\tilde{\lambda }_{jk}^{(1)}|\}>0$ immediately implies upon expanding $\widetilde{Q}_0(g)$,

4.51 $$\begin{aligned} e^{t\mathcal{L}_g}=e^{tg^2\widetilde{Q}_0\widetilde{\mathcal{L}}_1\widetilde{Q}_0}\widetilde{Q}_0+ \mathcal{O}(e^{-tg^2\eta }) +\mathcal{O}(g)+\mathcal{O}(g^3 t), \end{aligned}$$

where the constants in all $\mathcal{O}$ are uniform in $t\ge 0$ and *g* small. Finally,

$\widetilde{Q}_0\widetilde{\mathcal{L}}_1\widetilde{Q}_0=\tau _A\otimes \Phi _D\otimes \tau _B$ allows us to express the exponential in terms of that of $\Phi _D$. $\square$

## Construction of the Asymptotic State

We now turn to the determination of the state $\rho _0(g)\in \mathrm{Ker}\,\mathcal{L}_g$ where $\mathcal{L}_g=\mathcal{L}_0+g\mathcal{L}_1\in \mathcal{B}(\mathcal{B}(\mathcal{H}))$ given by a power series in *g*

5.1 $$\begin{aligned} \rho _0(g)=\rho _0+g\rho _1+g^2\rho _2+\cdots , \end{aligned}$$

where $\mathrm{tr}(\rho _0)=1$ and $\mathrm{tr}(\rho _j)=0$, $\forall j>0$. Expanding $\mathcal{L}_0(\rho _0(g))+g\mathcal{L}_1(\rho _0(g))\equiv 0$, and equating like powers of *g* we get

5.2 $$\begin{aligned} \mathcal{L}_0(\rho _0)&=0 \nonumber \\ \mathcal{L}_0(\rho _1)&+\mathcal{L}_1(\rho _0)=0 \nonumber \\ \mathcal{L}_0(\rho _2)&+\mathcal{L}_1(\rho _1)=0 \nonumber \\&\vdots \nonumber \\ \mathcal{L}_0(\rho _j)&+\mathcal{L}_1(\rho _{j-1})=0 \ \ \forall \ j\ge 1. \end{aligned}$$

The way to solve this set of equations, in principle, is as follows. Note that the spectral decomposition of $\mathcal{L}_0$ yields

5.3 $$\begin{aligned} \mathrm{Ker}\,\mathcal{L}_0=\mathrm{Ran}\,Q_0 \ \ \text{ and } \ \ \mathrm{Ran}\,\mathcal{L}_0=\mathrm{Ker}\,Q_0. \end{aligned}$$

The first equation is solved by picking a trace one element $R_0$ in $\mathrm{Ker}\,\mathcal{L}_0=Q_0(\mathcal{B}(\mathcal{H}))$, described in Proposition 3.7. The addition of any traceless vector $r_0\in \mathrm{Ker}\,\mathcal{L}_0$ yields an equally good solution for $\rho _0:=R_0+r_0$ at that order. The next equation amounts to solve $\mathcal{L}_0(R_1)=-\mathcal{L}_1(R_0+r_0)$ for $R_1$, a traceless matrix. This requires $\mathcal{L}_1(R_0+r_0)\in \mathrm{Ran}\,\mathcal{L}_0$. Since $\mathrm{Ran}\,\mathcal{L}_0=\mathrm{Ker}\,Q_0$, this is equivalent to $Q_0\mathcal{L}_1Q_0r_0=-Q_0\mathcal{L}_1R_0$, which determines $r_0=Q_0r_0$ up to the addition of an element of $\mathrm{Ker}\,Q_0\mathcal{L}_1Q_0$ ($Q_0\mathcal{L}_1Q_0$ viewed as an operator on $Q_0(\mathcal{B}(\mathcal{H}))$). Let us assume for the discussion here that $Q_0\mathcal{L}_1Q_0\ne 0$, *i.e.* $H_C=0$. This yields $R_1=-\mathcal{L}_0^{-1}(\mathcal{L}_1(R_0+r_0))$. Again, the addition of any traceless vector $r_1=Q_0r_1\in \mathrm{Ker}\,\mathcal{L}_0$ to that $R_1$ yields an equally good solution $\rho _1:=R_1+r_1$ to that equation. The next order requires $\mathcal{L}_1(r_1-\mathcal{L}_0^{-1}(\mathcal{L}_1(R_0+r_0)))\in \mathrm{Ran}\,\mathcal{L}_0$, which is equivalent to $Q_0\mathcal{L}_1Q_0r_1=Q_0\mathcal{L}_1\mathcal{L}_0^{-1}\mathcal{L}_1(R_0+r_0)$. This equation will then determine $r_0$ completely, under generic hypotheses, as we shall see. Then we proceed by induction.

The case $H_C\ne 0$ is slightly different, see Lemma 4.1, but is approached in the same spirit. We start by working out the first few steps and then give the general statements about this construction in Theorem 5.2 for $H_C=0$ and Theorem 5.4 for $H_C\ne 0$.

Again, the inverse of $\mathcal{L}_0$ on its range is the reduced resolvent $S_0=\mathcal{L}_0^{-1}({\mathbb {I}}- Q_0)=\mathcal{L}_0^{-1}|_{({\mathbb {I}}- Q_0)\mathcal{B}(\mathcal{H})}$. To express $S_0$, it is enough to consider the spectral decomposition $\mathcal{L}_0=\sum _{k>0}\lambda _k Q_k$, where $\lambda _k\ne 0$ and $Q_k$ are the spectral projectors corresponding to Proposition 3.7, while $\lambda _0=0$ corresponds to the projector $Q_0$.

### $H_C=0$

We consider here that $H_C=0$ and work under the spectral assumption **Spec(**$\overline{H}^{\, \tau }$**)** on the self-adjoint operator defined by (4.13). We first work out the orders $g^0$ and $g^1$ terms, *i.e.* $\rho _0$ and $\rho _1$, and then state an abstract result on the full perturbation series in Theorem 5.2.

The first equation yields $R_0=\tau _A\otimes \rho _C \otimes \tau _B$ where $\rho _C$ is a state. We choose $\rho _C=\frac{1}{n_C}{\mathbb {I}}_C$, and $r_0=\tau _A\otimes r_C^{(0)}\otimes \tau _B$ with any traceless $r_C^{(0)}$ can be added to that choice so that

5.4 $$\begin{aligned} \rho _0=\tau _A\otimes \rho _C^{(0)}\otimes \tau _B, \ \text{ with } \ \ \rho _C^{(0)}=\frac{1}{n_C}{\mathbb {I}}_C+ r_C^{(0)}. \end{aligned}$$

Then we compute $Q_0 \mathcal{L}_1(R_0+r_0)$:

5.5 $$\begin{aligned} Q_0(-i[H, R_0+r_0 ])=-i\tau _A\otimes [\overline{H}^{\, \tau }, \rho _C^{(0)}]\otimes \tau _B=-i\tau _A\otimes [\overline{H}^{\, \tau }, r_C^{(0)}]\otimes \tau _B. \end{aligned}$$

The condition to solve the equation for $R_1$ requires $r_C^{(0)}=\mathrm{Diag}_\tau (r_C^{(0)})$, where $\mathrm{Diag}_\tau (\cdot )$ extracts the diagonal part of $r_C^{(0)}$ in the normalised eigenbasis of $\overline{H}^{\, \tau }$. Thanks to our assumption, we set

5.6 $$\begin{aligned} R_1:=i\mathcal{L}_0^{-1}([H, R_0+r_0])=i\sum _{k>0}\lambda _k^{-1} Q_k([H, R_0+r_0]), \end{aligned}$$

which is traceless, since $R_1=({\mathbb {I}}-Q_0)R_1$, and self-adjoint if $r_C^{(0)}$ is. Next we look for $R_2$, which requires $Q_0(\mathcal{L}_1(R_1+r_1))=0$, where $r_1=Q_0(r_1)=\tau _A\otimes r_C^{(1)}\otimes \tau _B$:

5.7 $$\begin{aligned} Q_0([H,\{\mathcal{L}_0^{-1}(i[H, \tau _A\otimes \mathrm{Diag}_\tau (\rho _C^{(0)}) \otimes \tau _B])+\tau _A\otimes r_C^{(1)}\otimes \tau _B\}])=0. \end{aligned}$$

This is equivalent to the equation on $\mathcal{B}(\mathcal{H}_C)$

5.8 $$\begin{aligned} i\mathrm{tr}_{AB}\big (\big [H, \mathcal{L}_0^{-1}([H, \tau _A\otimes \mathrm{Diag}_\tau (\rho _C^{(0)}) \otimes \tau _B])\big ]\big )+ [\overline{H}^{\, \tau }, r_C^{(1)}]=0, \end{aligned}$$

where we note that $\mathrm{Diag}_\tau (r_C^{(1)})$ is arbitrary. Our hypotheses on $\overline{H}^{\, \tau }$ imply that

5.9 $$\begin{aligned} \mathrm{Ker}\,[\overline{H}^{\, \tau }, \cdot ]&=\{\rho _C\, | \, \rho _C=\mathrm{Diag}_\tau \rho _C \}, \end{aligned}$$

5.10 $$\begin{aligned} \mathrm{Ran}\,[\overline{H}^{\, \tau }, \cdot ]&=\{\rho _C\, | \, \rho _C=\mathrm{Offdiag}_\tau \rho _C\}. \end{aligned}$$

Now, assumption **Coup ** on *H* ensures (5.8) determines $\mathrm{Diag}_\tau r_C^{(0)}$ and $\mathrm{Offdiag}_\tau r_C^{(1)}$: Separating the diagonal from the offdiagonal parts, we have for the former

5.11 $$\begin{aligned} \Phi _D( \rho _C^{(0)})=0, \end{aligned}$$

which determines $\rho _C^{(0)}={\mathbb {I}}_C/n_C+\mathrm{Diag}_\tau r_C^{(0)}=\mathrm{Diag}_\tau (\rho _C^{(0)})$ fully since $\dim \mathrm{Ker}\,\Phi _D=1$, and thus $R_1$ as well. The offdiagonal part yields

5.12 $$\begin{aligned} \mathrm{Offdiag}_\tau r_C^{(1)}&= -i [\overline{H}^{\, \tau }, \cdot ]^{-1}\Big ( \mathrm{Offdiag}_\tau \mathrm{tr}_{AB}\big (\big [H, \mathcal{L}_0^{-1}([H, \tau _A\otimes \rho _C^{(0)} \otimes \tau _B])\big ]\big )\Big )\nonumber \\&=-i[\overline{H}^{\, \tau }, \cdot ]^{-1}\Big ( \mathrm{Offdiag}_\tau \Phi (\rho _C^{(0)})) \Big ) \end{aligned}$$

which fixes $\mathrm{Offdiag}_\tau r_C^{(1)}$ and leaves $\mathrm{Diag}_\tau r_C^{(1)}$ open for now.

At this point, the formula which defines $R_2$ makes sense,

5.13 $$\begin{aligned} R_2=i\mathcal{L}_0^{-1}([H, R_1+r_1])=i\sum _{k>0}\lambda _k^{-1} Q_k([H, R_1+r_1]), \end{aligned}$$

where $R_2$ depends parametrically on $\mathrm{Diag}_\tau r_C^{(1)}$. At order two, the contribution is $R_2+r_2$, where $r_2=Q_0(r_2)=\tau _A\otimes r_C^{(2)}\otimes \tau _B$ is arbitrary. The term $\mathrm{Diag}_\tau r_C^{(1)}$ is determined by the requirement that $Q_0(\mathcal{L}_1(R_2+r_2))=0$ necessary to solve for $R_3$, *i.e.*

5.14 $$\begin{aligned}&\mathrm{tr}_{AB}([H,\{\mathcal{L}_0^{-1}(i[H, R_1+\tau _A\otimes r_C^{(1)}\otimes \tau _B])+\tau _A\otimes r_C^{(2)}\otimes \tau _B\}])\nonumber \\&\quad =\mathrm{tr}_{AB}([H,\mathcal{L}_0^{-1}(i[H, R_1+\tau _A\otimes r_C^{(1)}\otimes \tau _B])+[\overline{H}^{\, \tau }, r_C^{(2)}]=0. \end{aligned}$$

Splitting this equation into its diagonal and offdiagonal parts, we get, making use of (4.29),

5.15 $$\begin{aligned}&\mathrm{Diag}_\tau \mathrm{tr}_{AB}([H,\mathcal{L}_0^{-1}(i[H, R_1+\tau _A\otimes \mathrm{Offdiag}_\tau r_C^{(1)}\otimes \tau _B])]+ \Phi _D (\mathrm{Diag}_\tau r_C^{(1)})=0, \end{aligned}$$

5.16 $$\begin{aligned}&\mathrm{Offdiag}_\tau \mathrm{tr}_{AB}([H,\mathcal{L}_0^{-1}(i[H, R_1+ \tau _A\otimes r_C^{(1)}\otimes \tau _B])]+[\overline{H}^{\, \tau }, r_C^{(2)}]=0. \end{aligned}$$

Using assumption **Coup** under the form: $\Phi _D$ is invertible on the subspace $\mathrm{Ran}\,\Phi _D=\mathrm{Diag}_\tau \mathcal{B}(\mathcal{H}_C)\cap \{\rho _C\, | \, \mathrm{tr}\rho _C=~0\}$, the first equation determines

5.17 $$\begin{aligned} \mathrm{Diag}_\tau r_C^{(1)}=-\Phi _D^{-1}(\mathrm{Diag}_\tau \mathrm{tr}_{AB}([H,\mathcal{L}_0^{-1}(i[H, R_1+\tau _A\otimes \mathrm{Offdiag}_\tau r_C^{(1)}\otimes \tau _B])]), \end{aligned}$$

so that $r_C^{(1)}$ is determined and therefore the second equation yields

5.18 $$\begin{aligned} \mathrm{Offdiag}_\tau r_C^{(2)}=-i[\overline{H}^{\, \tau }, \cdot ]^{-1}\Big ( \mathrm{Offdiag}_\tau \mathrm{tr}_{AB}\big (\big [H, \mathcal{L}_0^{-1}([H, R_1+\tau _A\otimes r_C^{(1)} \otimes \tau _B])\big ]\big )\Big ). \end{aligned}$$

Consequently, we can set

5.19 $$\begin{aligned} R_3=i\mathcal{L}_0^{-1}([H, R_2+r_2])=({\mathbb {I}}-Q_0)R_3. \end{aligned}$$

At this point, $\rho _0=R_0+r_0$, $\rho _1=R_1+r_1$ are known, as well as $R_2$, $\mathrm{Offdiag}_\tau r_C^{(2)}$ and $R_3$.

#### Remark 5.1

The fact that $\rho _C^{(0)}\in \mathrm{Ker}\,\Phi _D$ implies $\mathrm{tr}\rho _C^{(0)}\ne 0$, so the assumption that $\rho _C$ is a state in the initial step amounts to set a normalisation.

Let us formulate a general result that summarises the foregoing and guarantees the process can be pursued:

#### Theorem 5.2

Consider the QRM Lindbladian $\mathcal{L}_g$ (4.30) with $H_C=0$ under the assumptions **Spec(**$\overline{H}^{\, \tau }$**)** and **Coup**. Then there exists $g_0>0$ such that $\rho _0(g)$, the unique invariant state of $\mathcal{L}_g$, admits a convergent expansion

5.20 $$\begin{aligned} \rho _0(g)=\rho _0+g\rho _1+g^2\rho _2+\cdots , \end{aligned}$$

for all $g\in {\mathbb {C}}$ with $|g|<g_0$. We have,

5.21 $$\begin{aligned} \rho _0&=\tau _A\otimes \rho _C^{(0)} \otimes \tau _B, \ \text{ where } \ \rho _C^{(0)} \in \mathrm{Ker}\,\Phi _D \end{aligned}$$

see (4.29) and (5.4), and

5.22 $$\begin{aligned} \rho _j=R_j+\tau _A\otimes r_C^{(j)}\otimes \tau _B \end{aligned}$$

for all $j\ge 1$. Moreover, there exists a linear map $\mathcal{R}: \mathcal{B}(\mathcal{H})\rightarrow \mathcal{B}(\mathcal{H})\cap \{\rho _C\, | \, \mathrm{tr}\rho _C=~0\}$ such that $\rho _j=\mathcal{R}(\rho _{j-1}),$ where

5.23 $$\begin{aligned}&R_j=i\mathcal{L}_0^{-1}([H, \rho _{j-1}]), \end{aligned}$$

5.24 $$\begin{aligned}&\mathrm{Offdiag}_\tau r_C^{(j)}=-i[\overline{H}^{\, \tau }, \cdot ]^{-1}\Big ( \mathrm{Offdiag}_\tau \mathrm{tr}_{AB}\big (\big [H, \mathcal{L}_0^{-1}([H, \rho _{j-1}])\big ]\big )\Big ), \end{aligned}$$

5.25 $$\begin{aligned}&\mathrm{Diag}_\tau r_C^{(j)}=-\Phi _D^{-1}\big (\mathrm{Diag}_\tau \mathrm{tr}_{AB}([H,\mathcal{L}_0^{-1}(i[H, R_{j}+\tau _A\otimes \mathrm{Offdiag}_\tau r_C^{(j)}\otimes \tau _B])])\big ). \end{aligned}$$

Consequently, for $$|g|<g_0$$,

5.26 $$\begin{aligned} \rho _0(g)=({\mathbb {I}}-g\mathcal{R})^{-1}(\rho _0). \end{aligned}$$

#### Remark 5.3

0) Replacing $R_j$ and $\mathrm{Offdiag}_\tau r_C^{(j)}$ by their expression into (5.25) shows $\mathrm{Diag}_\tau r_C^{(j)}$ is linear in $\rho _{j-1}$ as well an yields the map $\mathcal{R}$.
i) Eq. (5.26) is equivalent to
  5.27 $$\begin{aligned} \rho _0(g)=\sum _{k=1}^{N} \left( \frac{M_k}{1-g\mu _k}+\sum _{l=1}^{m_k-1}\frac{g^l N_k^l}{(1-g\mu _k)^{l+1}}\right) (\rho _0), \end{aligned}$$
  where $\mu _k, M_k, N_k$ and $m_k$ are the eigenvalues, eigenprojectors, eigennilpotents and algebraic multiplicities appearing in the spectral decomposition of $\mathcal{R}=\sum _{k=1}^N\mu _k M_k+N_k$. Hence the radius of convergence is $g_0=1/\max _{1\le k\le N}(|\mu _k|)$.
ii) In case $\sigma (\mathcal{R})\cap {\mathbb {R}}_\pm ^*=\emptyset$, the steady state $\rho _0(g)$ is well defined for all $g\in {\mathbb {R}}_\pm ^*$.
iii) The iteration terminates if and only if $\mathcal{R}$ has a zero eigenvalue and $\rho _0$ belongs to the corresponding eigenspace; see Sect. 8 for examples.
iv) The restriction of the invariant state to $\mathcal{H}_C$ is given by $\mathrm{tr}_{AB}(\rho _0(g))=\rho _C^{(0)}+\sum _{j\ge 1}g^jr_C^{(j)}$.
v) We provide necessary and sufficient conditions in Proposition 6.1 for **Coup** to be satisfied in case $\mathcal{L}_0=\mathcal{D}$ and $H_C=0$.

#### Proof

Recall that $\dim \mathrm{Ker}\,\mathcal{L}_g=1$ is proven in Theorem 4.3.

We solve the higher orders equations for $\rho _j=R_j+r_j$ of (5.2) with

5.28 $$\begin{aligned} R_j=({\mathbb {I}}-Q_0)R_j, \ r_{j}=Q_0 r_{j}=\tau _A\otimes r_C^{(j)}\otimes \tau _B, \end{aligned}$$

for all *j* by induction. Let $j\ge 2$ and assume $R_{k}$, $r_{k}$ are given traceless matrices satisfying (5.28) for $1\le k\le j-1$ as well as

5.29 $$\begin{aligned}&R_{j}=i\mathcal{L}_0^{-1}([H,R_{j-1}+r_{j-1}]), \ \tau _A\otimes \mathrm{Offdiag}_\tau r_C^{(j)}\otimes \tau _B\ \text{ and } \ R_{j+1}=i\mathcal{L}_0^{-1}([H,R_{j}+r_{j}]). \end{aligned}$$

This is the situation we arrived at for $j=2$. Consider $Q_0(\mathcal{L}_1(R_{j+1}+r_{j+1}))=0$, a necessary condition to compute $R_{j+2}$, which yields

5.30 $$\begin{aligned} \mathrm{tr}_{AB}\big ([H,\mathcal{L}_0^{-1}(i[H, R_{j}+\tau _A\otimes r_C^{(j)}\otimes \tau _B])\big )+[\overline{H}^{\, \tau }, r_C^{(j)}]=0. \end{aligned}$$

Splitting the equation into its diagonal and offdiagonal parts gives

5.31 $$\begin{aligned}&\mathrm{Diag}_\tau \mathrm{tr}_{AB}\big ([H,\mathcal{L}_0^{-1}(i[H, R_{j}+\tau _A\otimes \mathrm{Offdiag}_\tau r_C^{(j)}\otimes \tau _B]\big )+\Phi _D(\mathrm{Diag}_\tau r_C^{(j)})=0, \end{aligned}$$

5.32 $$\begin{aligned}&\mathrm{Offdiag}_\tau \mathrm{tr}_{AB}\big ([H,\mathcal{L}_0^{-1}(i[H, R_{j}+\tau _A\otimes r_C^{(j)}\otimes \tau _B]\big )+[\overline{H}^{\, \tau }, r_C^{(j+1)}]=0. \end{aligned}$$

The first equation determines

5.33 $$\begin{aligned} \mathrm{Diag}_\tau r_C^{(j)}=-\Phi _D^{-1}\big (\mathrm{Diag}_\tau \mathrm{tr}_{AB}([H,\mathcal{L}_0^{-1}(i[H, R_{j}+\tau _A\otimes \mathrm{Offdiag}_\tau r_C^{(j)}\otimes \tau _B])])\big ), \end{aligned}$$

so that $r_C^{(j)}$ is fully determined and therefore the second equation yields

5.34 $$\begin{aligned} \mathrm{Offdiag}_\tau r_C^{(j+1)}=-i[\overline{H}^{\, \tau }, \cdot ]^{-1}\Big ( \mathrm{Offdiag}_\tau \mathrm{tr}_{AB}\big (\big [H, \mathcal{L}_0^{-1}([H, R_{j}+\tau _A\otimes r_C^{(j)} \otimes \tau _B])\big ]\big )\Big ). \end{aligned}$$

Consequently we can define

5.35 $$\begin{aligned} R_{j+2}=i\mathcal{L}_0^{-1}([H,R_{j+1}+r_{j+1}]), \end{aligned}$$

where $\mathrm{Diag}_\tau r_C^{(j+1)}$ remains free, while $r_j$ is determined. This finishes the proof of the induction. $\square$

### $H_C\ne$ 0

We consider here $H_C\ne 0$ and the necessary modifications to compute the series (5.1) due to the identities

5.36 $$\begin{aligned} Q_0 (\cdot )=\tau _A\otimes \mathrm{Diag}_C(\mathrm{tr}_{AB}(\cdot ))\otimes \tau _B \ \, \text{ and } \ \, Q_0\mathcal{L}_1Q_0\equiv 0. \end{aligned}$$

The first equation in (5.2) yields $\rho _0=Q_0 \rho _0=\tau _A\otimes \rho _C^{(0)} \otimes \tau _B$, where $\rho _C^{(0)}~\in ~\mathrm{Diag}_C\mathcal{B}(\mathcal{H}_C)$ is free. The condition to solve the second equation is $Q_0\mathcal{L}_1(\rho _0)=Q_0\mathcal{L}_1Q_0(\rho _0)=0$ which is trivially satisfied. Thus, writing $\rho _1=R_1+r_1$ with $R_1=({\mathbb {I}}-Q_0)\rho _1$ and $r_1=Q_0 \rho _1$, we can solve partially the equation setting

5.37 $$\begin{aligned} R_1=-\mathcal{L}_0^{-1}\mathcal{L}_1(\rho _0). \end{aligned}$$

The next equation $\mathcal{L}_0(\rho _2)=-\mathcal{L}_1(\rho _1)$ requires $Q_0\mathcal{L}_1(R_1)+Q_0\mathcal{L}_1 (r_1)=0$. Thanks to $r_1=Q_0 r_1$ and the identity (5.36), this equation reduces to

5.38 $$\begin{aligned} Q_0\mathcal{L}_1\mathcal{L}_0^{-1}\mathcal{L}_1 Q_0(\rho _0)=0, \end{aligned}$$

where we used the expression for $R_1$ and $\rho _0=Q_0 \rho _0$. Thanks to assumption **Coup** for $H_C\ne 0$, this determines $\rho _0=\tau _A\otimes \mathrm{Diag}_C\rho _C^{(0)} \otimes \tau _B$ since (5.38) is equivalent to

5.39 $$\begin{aligned} \rho _C^{(0)} \in \mathrm{Ker}\,\Phi _D, \ \text{ where } \ \dim \mathrm{Ker}\,\Phi _D=1. \end{aligned}$$

Thus $R_1$ is now determined, while the traceless part $r_1=\tau _A\otimes \mathrm{Diag}_C r_C^{(1)}\otimes \tau _B$ is not. With the familiar decomposition $\rho _2=R_2+r_2$ with respect to the projector $Q_0$, we set

5.40 $$\begin{aligned} R_2=-\mathcal{L}_0^{-1}\mathcal{L}_1(R_1+r_1) \end{aligned}$$

and turn to the equation for $\rho _3=R_3+r_3$: $\mathcal{L}_0(\rho _3)=\mathcal{L}_0(R_3)=-\mathcal{L}_1(\rho _2)$. It requires $Q_0\mathcal{L}_1(R_2+r_2)=Q_0\mathcal{L}_1(R_2)=0$, where we used (5.36) and $r_2=Q_0 r_2$. With (5.40), this is equivalent to

5.41 $$\begin{aligned} Q_0\mathcal{L}_1\mathcal{L}_0^{-1}\mathcal{L}_1 Q_0(r_1)=-Q_0\mathcal{L}_1\mathcal{L}_0^{-1}\mathcal{L}_1(R_1)=-\tau _A\otimes \mathrm{Diag}_C \mathrm{tr}_{AB}(\mathcal{L}_1\mathcal{L}_0^{-1}\mathcal{L}_1(R_1))\otimes \tau _B, \end{aligned}$$

where $\mathrm{tr}\mathcal{L}_1\mathcal{L}_0^{-1}\mathcal{L}_1(R_1)=0$, since $\mathcal{L}_1(\cdot )=-i[H, \cdot ]$. Thanks to **Coup**, we can thus determine $r_1=\tau _A\otimes \mathrm{Diag}_C r_C^{(1)}\otimes \tau _B$ uniquely in terms of $\Phi _D$

5.42 $$\begin{aligned} r_C^{(1)}=\Phi _D^{-1}\Big (\mathrm{Diag}_C \mathrm{tr}_{AB}\big \{\big [H,\mathcal{L}_0^{-1}([H, R_1]\big ]\big \} \Big ). \end{aligned}$$

In turn $R_2$ is fully determined while $r_2=\tau _A\otimes \mathrm{Diag}_C r_C^{(2)}\otimes \tau _B$ remains to be computed, and

5.43 $$\begin{aligned} R_3=-\mathcal{L}_0^{-1}\mathcal{L}_1(R_2+r_2). \end{aligned}$$

From there on we can iterate the process to get the equivalent of Theorem 5.2 in the case $H_C\ne 0$. The proof being similar and simpler, we omit it.

#### Theorem 5.4

Consider the QRM Lindbladian $\mathcal{L}_g$ (4.30) with $H_C\ne 0$ under the assumptions **Spec(**$\overline{H}^{\, \tau }$**)** and **Coup**. Then there exists $g_0>0$ such that $\rho _0(g)$, the unique invariant state of $\mathcal{L}_g$, admits a convergent expansion

5.44 $$\begin{aligned} \rho _0(g)=\rho _0+g\rho _1+g^2\rho _2+\cdots , \end{aligned}$$

for all $g\in {\mathbb {C}}$ with $|g|<g_0$. We have,

5.45 $$\begin{aligned} \rho _0&=\tau _A\otimes \rho _C^{(0)} \otimes \tau _B, \ \text{ where } \ \rho _C^{(0)} \in \mathrm{Ker}\,\Phi _D \end{aligned}$$

see (4.29) and (5.39), and $\rho _j=R_j+\tau _A\otimes r_C^{(j)}\otimes \tau _B$ for all $j\ge 1$, with $r_C^{(j)}=\mathrm{Diag}_C ( r_C^{(j)})$. Moreover, there exists a linear map $\mathcal{R}: \mathcal{B}(\mathcal{H})\rightarrow \mathcal{B}(\mathcal{H})\cap \{\rho _C\, | \, \mathrm{tr}\rho _C=0\}$ such that $\rho _j=\mathcal{R}(\rho _{j-1}),$ where

5.46 $$\begin{aligned} R_j&=i\mathcal{L}_0^{-1}([H, \rho _{j-1}]), \end{aligned}$$

5.47 $$\begin{aligned} r_C^{(j)}&=\Phi _D^{-1}\big (\mathrm{Diag}_C \mathrm{tr}_{AB}([H,\mathcal{L}_0^{-1}([H, R_{j}])])\big )\nonumber \\&=i\Phi _D^{-1}\big (\mathrm{Diag}_C \mathrm{tr}_{AB}([H,\mathcal{L}_0^{-1}([H, \mathcal{L}_0^{-1}([H, \rho _{j-1}])])])\big ). \end{aligned}$$

Consequently, for $$|g|<g_0$$,

5.48 $$\begin{aligned} \rho _0(g)=({\mathbb {I}}-g\mathcal{R})^{-1}(\rho _0). \end{aligned}$$

#### Remark 5.5

0) Remarks (i), (ii), (iii) below Theorem 5.2 remain in force here.
i) The map $\mathcal{R}$ can be expressed as
  5.49 $$\begin{aligned} R_{j}&=-\mathcal{L}_0^{-1}\mathcal{L}_1(\rho _{j-1}), \nonumber \\ r_C^{(j)}&=-\Phi _D^{-1}\big (\mathrm{tr}_{AB}\big \{ Q_0\mathcal{L}_1\mathcal{L}_0^{-1}\mathcal{L}_1 (R_{j})\big \}\big )=\Phi _D^{-1}\big (\mathrm{tr}_{AB}\big \{ Q_0\mathcal{L}_1\mathcal{L}_0^{-1}\mathcal{L}_1 \mathcal{L}_0^{-1}\mathcal{L}_1(\rho _{j-1})\big \}\big ) \end{aligned}$$
  so that
  5.50 $$\begin{aligned} \rho _j=\Big (-\mathcal{L}_0^{-1}\mathcal{L}_1(\cdot )+\tau _A\otimes \Phi _D^{-1}\big (\mathrm{tr}_{AB}\big \{ Q_0\mathcal{L}_1\mathcal{L}_0^{-1}\mathcal{L}_1 \mathcal{L}_0^{-1}\mathcal{L}_1(\cdot )\big \}\big ) \otimes \tau _B\Big )(\rho _{j-1}). \end{aligned}$$

## No Leading order Hamiltonian Drive

We consider here the case where $H_A=H_B=H_C=0$ on their respective spaces, so that $\mathcal{L}_0=\mathcal{D}$ with $\tau _A$ and $\tau _B$ arbitrary, while $\mathcal{L}_1=-i[H,\, \cdot \, ]$ with *H* arbitrary as well. This allows us to keep things relatively simple, while retaining a certain level of generality, since the dimensions of the different Hilbert spaces are arbitrary as well.

Let us consider the hypothesis **Coup** in this simplified setup, assuming **Spec(**$\overline{H}^{\, \tau }$**)** holds. Recall that $\{\varphi _j^\tau \}_{1\le j\le n_C}$ denotes the normalized eigenbasis of $\overline{H}^{\, \tau }$ with respect to which the projectors $\mathrm{Diag}_\tau$ and $\mathrm{Offdiag}_\tau$ are defined, and set $P_j^\tau =|\varphi _j^\tau \rangle \langle \varphi _j^\tau |$. Given the definition (4.29) of $\Phi _D$, we need to compute for all $j,k\in \{1,\dots , n_C\}$

6.1 $$\begin{aligned} (\Phi _D)_{jk}:=\mathrm{tr}\big \{({\mathbb {I}}_A\otimes P_j^\tau \otimes {\mathbb {I}}_B)\big ([H,\mathcal{L}_0^{-1}( [H,\tau _A\otimes P_k^\tau \otimes \tau _B]\big )\big \}. \end{aligned}$$

Thanks to Proposition 3.4, we can express $\mathcal{L}_0^{-1}=\mathcal{D}^{-1}$ in a compact way. Let $\tilde{\rho }_0\in \mathcal{B}(\mathcal{H})$ such that $\mathrm{tr}_{AB}(\tilde{\rho }_0)=0$, so that $Q_0(\tilde{\rho }_0)=0$. Thus

6.2 $$\begin{aligned} \mathcal{D}^{-1}(\tilde{\rho }_0)=\frac{-1}{\gamma _A+\gamma _B}\left\{ \tilde{\rho }_0+\frac{\gamma _A}{\gamma _B} \tau _A \otimes \mathrm{tr}_A(\tilde{\rho }_0)+\frac{\gamma _B}{\gamma _A}\mathrm{tr}_B(\tilde{\rho }_0)\otimes \tau _B\right\} \end{aligned}$$

Therefore, introducing

6.3 $$\begin{aligned} \overline{H}^{\, \tau _A}&=\mathrm{tr}_{A}(H(\tau _A\otimes {\mathbb {I}}_C \otimes {\mathbb {I}}_B))=\mathrm{tr}_{A}((\tau _A\otimes {\mathbb {I}}_C \otimes {\mathbb {I}}_B)H)\in \mathcal{B}(\mathcal{H}_C\otimes \mathcal{H}_B), \end{aligned}$$

6.4 $$\begin{aligned} \overline{H}^{\, \tau _B}&=\mathrm{tr}_{B}(H({\mathbb {I}}_A\otimes {\mathbb {I}}_C \otimes \tau _B))=\mathrm{tr}_{B}(({\mathbb {I}}_A\otimes {\mathbb {I}}_C \otimes \tau _B)H)\in \mathcal{B}(\mathcal{H}_A\otimes \mathcal{H}_C) \end{aligned}$$

and making use of $\mathrm{tr}_{AB} [H,\tau _A\otimes P_k^\tau \otimes \tau _B]=0$, a straightforward computation yields

6.5 $$\begin{aligned}&[H,\mathcal{L}_0^{-1}( [H,\tau _A\otimes P_k^\tau \otimes \tau _B])]=-\frac{1}{\gamma _A+\gamma _B} [H,[H,\tau _A\otimes P_k^\tau \otimes \tau _B]]\nonumber \\&\quad -\frac{\gamma _A/\gamma _B}{\gamma _A+\gamma _B}[H,\tau _A\otimes [\overline{H}^{\, \tau _A}, P_k^\tau \otimes \tau _B]]-\frac{\gamma _B/\gamma _A}{\gamma _A+\gamma _B}[H,[\overline{H}^{\, \tau _B},\tau _A\otimes P_k^\tau ]\otimes \tau _B]. \end{aligned}$$

Then we note using the cyclicity of the trace that

6.6 $$\begin{aligned}&\mathrm{tr}\big \{({\mathbb {I}}_A\otimes P_j^\tau \otimes {\mathbb {I}}_B)\big ([H,[H,\tau _A\otimes P_k^\tau \otimes \tau _B]])\big \} \nonumber \\&\quad =2\big (\delta _{jk}\mathrm{tr}(H(\tau _A\otimes P_k^\tau \otimes \tau _B)H) -\mathrm{tr}(({\mathbb {I}}_A\otimes P_j^\tau \otimes {\mathbb {I}}_B)H(\tau _A\otimes P_k^\tau \otimes \tau _B)H)\big ) \end{aligned}$$

where the operator in the first trace reads

6.7 $$\begin{aligned} \big ((\tau _A^{1/2}\otimes P_k^\tau \otimes \tau _B^{1/2})H\big )^*(\tau _A^{1/2}\otimes P_k^\tau \otimes \tau _B^{1/2})H\ge 0, \end{aligned}$$

while the second trace yields the *jj* element of its partial $\mathrm{tr}_{AB}$. Hence,

6.8 $$\begin{aligned}&\mathrm{tr}\big \{({\mathbb {I}}_A\otimes P_j^\tau \otimes {\mathbb {I}}_B)\big ([H,[H,\tau _A\otimes P_k^\tau \otimes \tau _B]])\big \}\nonumber \\&\quad =2 \left\{ \begin{matrix} - \mathrm{tr}_{AB} (H(\tau _A\otimes P_k^\tau \otimes \tau _B)H)_{jj}\le 0&{} \text{ if } \ j\ne k \\ \sum _{l\ne k}\mathrm{tr}_{AB} (H(\tau _A\otimes P_k^\tau \otimes \tau _B)H)_{ll}\ge 0&{} \text{ if } \ j = k \end{matrix}\right. . \end{aligned}$$

Similar considerations can be made for the traces of the other two operators in (6.5):

6.9 $$\begin{aligned} \mathrm{tr}\big \{({\mathbb {I}}_A\otimes P_j^\tau&\otimes {\mathbb {I}}_B)\big ([H,[\overline{H}^{\, \tau _B},\tau _A\otimes P_k^\tau ]\otimes \tau _B]\big )\big \} = \mathrm{tr}\big \{({\mathbb {I}}_A\otimes P_j^\tau )\big ([\overline{H}^{\, \tau _B},[\overline{H}^{\, \tau _B},\tau _A\otimes P_k^\tau ]])\big \} \nonumber \\&=2\big (\delta _{jk}\mathrm{tr}(\overline{H}^{\, \tau _B}(\tau _A\otimes P_k^\tau )\overline{H}^{\, \tau _B}) -\mathrm{tr}(({\mathbb {I}}_A\otimes P_j^\tau )\overline{H}^{\, \tau _B}(\tau _A\otimes P_k^\tau )\overline{H}^{\, \tau _B})\big )\nonumber \\&=2 \left\{ \begin{matrix} - \mathrm{tr}_{A} (\overline{H}^{\, \tau _B}(\tau _A\otimes P_k^\tau )\overline{H}^{\, \tau _B})_{jj}\le 0&{} \text{ if } \ j\ne k \\ \sum _{l\ne k}\mathrm{tr}_{A} (\overline{H}^{\, \tau _B}(\tau _A\otimes P_k^\tau )\overline{H}^{\, \tau _B})_{ll}\ge 0&{} \text{ if } \ j = k \end{matrix}\right. , \end{aligned}$$

and

6.10 $$\begin{aligned} \mathrm{tr}\big \{({\mathbb {I}}_A\otimes P_j^\tau&\otimes {\mathbb {I}}_B)\big ([H,\tau _A\otimes [\overline{H}^{\, \tau _A},P_k^\tau \otimes \tau _B]]\big )\big \} = \mathrm{tr}\big \{(P_j^\tau \otimes {\mathbb {I}}_B)\big ([\overline{H}^{\, \tau _A},[\overline{H}^{\, \tau _A},P_k^\tau \otimes \tau _B]])\big \} \nonumber \\&=2\big (\delta _{jk}\mathrm{tr}(\overline{H}^{\, \tau _A}(P_k^\tau \otimes \tau _B)\overline{H}^{\, \tau _A}) -\mathrm{tr}((P_j^\tau \otimes {\mathbb {I}}_A)\overline{H}^{\, \tau _A}(P_k^\tau \otimes \tau _B)\overline{H}^{\, \tau _A})\big )\nonumber \\&=2 \left\{ \begin{matrix} - \mathrm{tr}_{B} (\overline{H}^{\, \tau _A}(P_k^\tau \otimes \tau _B)\overline{H}^{\, \tau _A})_{jj}\le 0&{} \text{ if } \ j\ne k \\ \sum _{l\ne k}\mathrm{tr}_{B} (\overline{H}^{\, \tau _B}(P_k^\tau \otimes \tau _B)\overline{H}^{\, \tau _B})_{ll}\ge 0&{} \text{ if } \ j = k \end{matrix}\right. . \end{aligned}$$

Defining for $1\le k \le n_C$ the non negative operator $h(k)\in \mathcal{B}(\mathcal{H}_C)$ by

6.11 $$\begin{aligned} h(k)=&\frac{2}{\gamma _A+\gamma _B}\mathrm{tr}_{AB} (H(\tau _A\otimes P_k^\tau \otimes \tau _B)H)\nonumber \\&+\frac{2\gamma _A/\gamma _B}{\gamma _A+\gamma _B}\mathrm{tr}_{B} (\overline{H}^{\, \tau _A}(P_k^\tau \otimes \tau _B)\overline{H}^{\, \tau _A}) +\frac{2\gamma _B/\gamma _A}{\gamma _A+\gamma _B}\mathrm{tr}_{A} (\overline{H}^{\, \tau _B}(\tau _A\otimes P_k^\tau )\overline{H}^{\, \tau _B}), \end{aligned}$$

we eventually obtain

6.12 $$\begin{aligned} (\Phi _D)_{jk}= \left\{ \begin{matrix} - h(k)_{jj}&{}\ge 0&{} \text{ if } \ j\ne k \\ +\sum _{l\ne k} h(k)_{ll}&{}\ne 0&{} \text{ if } \ j = k \end{matrix}\right. , \end{aligned}$$

where $\Phi _D$ is viewed as a matrix on ${\mathbb {C}}^{n_C}$, and any diagonal matrix $r=\sum _{k=1}^{n_C}{r_k}P_k^\tau \in \mathrm{Diag}_\tau \mathcal{B}(\mathcal{H}_C)$ is viewed as a vector $$\begin{pmatrix} r_1&r_2&\cdots&r_{n_C}\end{pmatrix}^t$$ of ${\mathbb {C}}^{n_C}$.

We provide a necessary and sufficient condition on the coupling Hamiltonian *H* in terms of the diagonal matrix elements of *h*(*k*), $1\le k\le n_C$ for assumption **Coup** to hold, *i.e.* that $\Phi _D$ restricted to diagonal traceless matrices is invertible.

### Proposition 6.1

Assume $\mathcal{L}_0=\mathcal{D}$, $\mathcal{L}_1=-i[H,\cdot \, ]$ and consider the non negative operators $\{h(k)\}_{1\le k\le n_C}$ defined by (6.11). Assumption **Coup** holds if and only if there exists $j\in \{1, \dots , n_C\}$ such that $h_{jj}(k)>0$ for all $1\le k\ne j \le n_C$.

### Remark 6.2

i) Since *h*(*k*) is a sum of non negative operators, it is sufficient to check the condition on any of its constituants.
ii) Explicit computations show that for $\dim \mathcal{H}_C=2$, assumption **Coup** holds as soon as $\Phi _D\ne 0$, while for $\dim \mathcal{H}_C=3$ it is true if $h_r(j)h_s(k)>0$ for some $1\le j\ne k\le 3$, $r\ne j$, $s\ne k$ and $(r,s)\ne (k,j)$.

### Proof

Within the framework introduced above we identify $\Phi _D$ with its matrix $(\Phi _D)_{jk}$. We need to show it admits zero as a simple eigenvalue, which amounts to showing that $\mathrm{Rank}\,\Phi _D=n_C-1$.

We use the short hand notations $h_j(k)=h(k)_{jj}\ge 0$ for $j\ne k$ and $h_j(j)=\sum _{k\ne j}h_k(j)\ge 0$ to express the matrix elements of $-\Phi _D$. The proof follows once we establish the following Lemma $\square$

### Lemma 6.3

Consider ${\mathfrak {h}}\in M_n({\mathbb {R}})$ given by

6.13 $$\begin{aligned} {\mathfrak {h}} = \begin{pmatrix} h_1(1) &{} -h_1(2) &{} \cdots &{} -h_1(n) \\ -h_2(1) &{} h_2(2) &{} &{} -h_2(n) \\ \vdots &{} &{}\ddots &{} \vdots \\ -h_n(1) &{} -h_n(2) &{} \cdots &{} h_n(n) \end{pmatrix}, \ \text{ where } \ \left\{ \begin{matrix}h_j(k)\ge 0 \ \ \ \ \ \, \text{ for } \ j\ne k \\ h_j(j)=\sum _{k\ne j}h_k(j) \ge 0 \end{matrix}\right. . \end{aligned}$$

Then, $\mathrm{Rank}\,\, {\mathfrak {h}}=n-1$ if and only if $\ \exists \ 1\le j\le n$ such that $h_j(k)>0$, $\forall \ 1\le k \ne j \le n$.

### Remark 6.4

It is possible that $\mathrm{Rank}\,\, {\mathfrak {h}}=n-1$ and one diagonal element $h_j(j)=0$, in which case ${\mathfrak {h}}e_j=0$, where $e_j$ is the $j^{\text{ th }}$ canonical basis vector of ${\mathbb {C}}^n$.

We can associate to ${\mathfrak {h}}$ a stochastic matrix ${\mathfrak {p}}$ the elements of which are6.14such that $x\in {\mathbb {C}}^n$ satisfies ${\mathfrak {h}}x=0$ iff ${\mathfrak {p}}^ty=y$, where $y=\mathrm{Diag}({\mathfrak {h}})x\in {\mathbb {C}}^n$, if $h_k(k)>0$ for all *k*. Hence, if $\mathrm{Rank}\,\, {\mathfrak {h}}=n-1$, the components of *x* can all be chosen to be non negative, by Perron Frobenius theorem.

However $\mathfrak {p}$ is not necessarily irreducible as one sees from the example $${\mathfrak {h}} = \begin{pmatrix} 1 &{} 0 &{} 0 \\ -1 &{} 1 &{} -1 \\ 0&{} -1 &{} 1 \end{pmatrix}$$ with $\sigma ({\mathfrak {h}} )=\{0,1,2\}$ that admits the non strictly positive eigenvector $$\begin{pmatrix} 0&1&1 \end{pmatrix}^T$$ in its kernel

### Proof

We know $0\in \sigma ({\mathfrak {h}})$ and by Jacobi’s formula,

6.15 $$\begin{aligned} \frac{d}{dz}\det ({\mathfrak {h}}-z)|_{z=0}=\mathrm{tr}\ \text{ com}^t({\mathfrak {h}})=\sum _{j=1}^n\det \hat{\mathfrak {h}}_{jj}, \end{aligned}$$

where $\text{ com }(A)$ is the comatrix of *A* and $\hat{A}_{jk}$ is obtained by deleting the $j^\mathrm{th}$ row and $k^\mathrm{th}$ column of *A*. In our case

6.16 $$\begin{aligned} \hat{\mathfrak {h}}_{jj}=\begin{pmatrix} h_1(1) &{}\cdots &{} -h_1(j-1) &{} -h_1(j+1) &{} \cdots &{} -h_1(n) \\ \vdots &{} \ddots &{} &{}\vdots &{} &{} \vdots \\ -h_{j-1}(1) &{} \cdots &{} h_{j-1}(j-1) &{} -h_{j-1}(j+1) &{} &{}-h_{j-1}(n) \\ -h_{j+1}(1) &{} &{} -h_{j+1}(j-1)&{} h_{j+1}(j+1)&{} &{} -h_{j+1}(n) \\ \vdots &{} &{} \vdots &{} &{}\ddots &{} \vdots \\ -h_n(1) &{} \cdots &{}-h_n(j-1) &{} -h_n(j+1) &{}\cdots &{} h_n(n) \end{pmatrix} \end{aligned}$$

is real valued so that $\sigma (\hat{\mathfrak {h}}_{jj})=\overline{\sigma (\hat{\mathfrak {h}}_{jj})}$. Moreover, by definition, for all $k\ne j$

6.17 $$\begin{aligned} h_k(k)=\sum _{l\ne k}h_l(k)\ge {\mathop {\mathop {\sum }\limits _{l\ne k}}\limits _{l\ne j}}h_l(k), \end{aligned}$$

so that by Gershgorin Theorem

6.18 $$\begin{aligned} \sigma (\hat{\mathfrak {h}}_{jj})\subset \bigcup _{k\ne j}\Big \{z\in {\mathbb {C}}\, | \, |z-h_k(k)|\le {\mathop {\mathop {\sum }\limits _{l\ne k}}\limits _{l\ne j}} h_l(k)\Big \}\equiv \bigcup _{k\ne j} G_k \end{aligned}$$

where the circle $G_k$ centered at $h_k(k)$ of radius ${\mathop {\mathop {\sum }\limits _{l\ne k}}\limits _{l\ne j}} h_l(k)$ intersects the imaginary axis if and only if $h_j(k)=0$, in which case the intersection reduces to the origin. Since the determinant of $\hat{\mathfrak {h}}_{jj}$ is the product of its complex conjugate eigenvalues, (6.18) yields

6.19 $$\begin{aligned} \det \hat{\mathfrak {h}}_{jj}\ge 0, \ \text{ with } \text{ equality } \text{ iff } \ \exists \ k\ne j \ \text{ s.t. } \ h_j(k)=0. \end{aligned}$$

Therefore

6.20 $$\begin{aligned} \sum _{j=1}^n \det \hat{\mathfrak {h}}_{jj}\ge 0, \ \text{ with } \text{ equality } \text{ iff } \ \forall \ 1\le j\le n, \ \exists \ k\ne j \ \text{ s.t. } \ h_j(k)=0. \end{aligned}$$

$$\square $$

This ends the proof of the Proposition.

### Emergence of a Classical Markov Process

Coming back to Corollary 4.7, we know that for times s.t. $0\le t\le \frac{1}{F+\epsilon } |\ln (g)|/g^2$, the evolution semigroup $e^{t(\mathcal{D}(\cdot )-ig[H,\cdot ])}$ can be approximated by

6.21 $$\begin{aligned} e^{tg^2\Phi _D}: \mathrm{Diag}_\tau \mathcal{B}(\mathcal{H}_C)\rightarrow \mathrm{Diag}_\tau \mathcal{B}(\mathcal{H}_C). \end{aligned}$$

In the case at hand, $\Phi _D$ is expressed in the orthonormal basis $\{ |\varphi _j^\tau \rangle \langle \varphi _j^\tau |\}_{1\le j\le n_C}$ as the matrix (6.12) denoted by $\mathfrak {h}$ in Lemma 6.3. The negative of the transpose ${\mathfrak {h}}^T$ of $\mathfrak {h}$ is thus a *transition rate matrix* or *Q-matrix*, associated to a classical continuous time Markov chain with finitely many states, see [24]. Therefore we can associate to our quantum problem $\dot{\rho }= \mathcal{D}(\rho )-ig[H,\rho ]$ a classical continuous time Markov chain $(X_t)_{t\ge 0}$ on the state space $\{|\varphi _j^\tau \rangle \langle \varphi _j^\tau |\}_{1\le j \le n_C}$ identified with $\{1,2,\dots , n\}$ with $n=n_C$, as follows.

Let us recall the general framework. The Markov process $(X_t)_{t\ge 0}$ is characterised by the probability to find the process in state *j* at time $t\ge 0$, given the process at time 0 is in state *i*, is denoted by

6.22 $$\begin{aligned} p_{ij}(t)= {\mathbb {P}}(X_t=j|X_0=i), \ \ i,j\in \{1,2,\dots , n\}. \end{aligned}$$

These transition probabilities $P(t)=(p_{ij}(t))_{1\le i,j\le n}$ are solutions to the matrix form forward and backward equations

6.23 $$\begin{aligned} P'(t)=P(t)Q, \ \ {P(0)={\mathbb {I}}} \ \ \Leftrightarrow \ \ P'(t)^T=Q^TP^T(t), \ \ {P(0)={\mathbb {I}}}, \end{aligned}$$

where $Q=(q_{ij})_{1\le i,j\le n}$ is a transition rate matrix such that $q_{ii}\le 0$, $q_{ij}\ge 0$ and $\sum _{j=1}^nq_{ij}=0$. Hence, with the identification $Q=-{\mathfrak {h}}^T$ we get the following interpretation

#### Theorem 6.5

Consider $\mathcal{L}_g(\cdot )=\mathcal{D}(\cdot )-ig[H,\cdot ]$ under assumptions **Spec(**$\overline{H}^{\, \tau }$**)** and **Coup**. Then, the operator $e^{tg^2\Phi _D}$ arising in the approximation of $e^{t\mathcal{L}_g}$ provided in (4.44), describes a (rescaled) continuous time Markov process $(X_t)_{t\ge 0}$ on the state space $\{|\varphi _j^\tau \rangle \langle \varphi _j^\tau |\}_{1\le j \le n_C}\equiv \{1,\dots , n \}$ such that for all $S\ge 0$,

6.24 $$\begin{aligned} {\mathbb {P}}(X_s=j|X_0=i)= \mathrm{tr}_C\big \{ |\varphi _i^\tau \rangle \langle \varphi _i^\tau | e^{s \Phi _D}(|\varphi _j^\tau \rangle \langle \varphi _j^\tau |)\big \}. \end{aligned}$$

#### Remark 6.6

Therefore, for any $S \ge 0$, the transpose of $e^{s \Phi _D}$ is a stochastic matrix.

Let us note that appearance of a classical Markov process on the eigenstates of the leading order driving Hamiltonian within the derivation of Lindblad generators for open quantum systems is well known. By contrast, in absence of leading order driving Hamiltonian, the state space of the Markov process into play is determined by the eigenstates of the averaged first order Hamiltonian $\overline{H}^\tau$, which takes into account the effects of the reset matrices.

Finally, let us address the computation of the order $g^2$ corrections (4.32) of the simple eigenvalues $\lambda _{jk}(g)$ of $\mathcal{L}_g(\cdot )=\mathcal{D}(\cdot )-ig[H,\cdot ]$ given by

6.25 $$\begin{aligned} \tilde{\lambda }_{jk}^{(1)}=\mathrm{tr}\Big \{({\mathbb {I}}_A\otimes |\varphi _k^\tau \rangle \langle \varphi _j^\tau |\otimes {\mathbb {I}}_B)\big [H,\mathcal{L}_0^{-1}([H,\tau _A\otimes |\varphi _j^\tau \rangle \langle \varphi _k^\tau |\otimes \tau _B])\big ]\Big \}. \end{aligned}$$

We prove in Appendix that

#### Proposition 6.7

Consider $\mathcal{L}_g(\cdot )=\mathcal{D}(\cdot )-ig[H,\cdot ]$ under assumptions **Spec(**$\overline{H}^{\, \tau }$**)** and **Coup**. Then, the eigenvalues $\lambda _{jk}(g)$ of $\mathcal{L}_g$, see Proposition 4.5, satisfy

6.26 $$\begin{aligned} \mathfrak {R}\lambda _{jk}(g) \le -g^2\frac{\gamma _A^2+\gamma _A\gamma _B+\gamma _B^2}{\gamma _A\gamma _B(\gamma _A+\gamma _B)}(e_j^\tau -e_k^\tau )^2+\mathcal{O}(g^3) \end{aligned}$$

#### Remark 6.8

Actually, we show that $\mathfrak {R}\tilde{\lambda }_{jk}^{(1)}$ is upper bounded by a sum of non positive explicit contributions. Hence one can decrease the contributions stemming from these eigenvalues in the approximations of the dynamics shown in Corollary 4.7 by assuming the coupling Hamiltonian *H* makes the lower bounds of Lemma 9.1 below large enough.

## Example on ${\mathbb {C}}^2\otimes {\mathbb {C}}^N\otimes {\mathbb {C}}^2$

We present here an example where the two parts of the Hilbert space on which the dissipator acts non trivially are both ${\mathbb {C}}^2=\mathcal{H}_A=\mathcal{H}_B$, while the central part $\mathcal{H}_C={\mathbb {C}}^N$, with *N* arbitrary. The orthonormal bases of $\mathcal{H}_A$, $\mathcal{H}_B$ and $\mathcal{H}_C$ are denoted respectively by $\{|g\rangle , |e\rangle \}$, $\{|\downarrow \rangle , |\uparrow \rangle \}$ and $\{\varphi _j\}_{j=1}^N$. The reset states associated with rates $\gamma _A, \gamma _B>0$ are

7.1 $$\begin{aligned} \tau _A=t_A |g\rangle \langle g|+(1-t_A)|e\rangle \langle e|, \ \ \tau _B=t_B |\downarrow \rangle \langle \downarrow |+(1-t_B)|\uparrow \rangle \langle \uparrow |, \end{aligned}$$

where $0<t_A,t_B <1$. We consider again a case without leading order Hamiltonian drive, that is $H_A=H_B=H_C=0$, while the order *g* Hamiltonian reads

7.2 $$\begin{aligned} H&=H_\alpha \otimes {\mathbb {I}}_B+{\mathbb {I}}_A\otimes H_\beta , \ \text{ where }\nonumber \\ H_\alpha&=\sum _{j=1}^N a_j^{(g)}|g\otimes \varphi _j\rangle \langle g\otimes \varphi _j |+ a_j^{(e)}|e\otimes \varphi _j\rangle \langle e\otimes \varphi _j |+\sum _{k=1}^N\alpha _k|g\otimes \varphi _1 \rangle \langle e\otimes \varphi _k | + \text{ h.c. }\nonumber \\ H_\beta&=\sum _{j=1}^N b_j^{(\downarrow )}|\varphi _j\otimes \downarrow \rangle \langle \varphi _j\otimes \downarrow |+ b_j^{(\uparrow )}|\varphi _j\otimes \uparrow \rangle \langle \varphi _j\otimes \uparrow |+\sum _{k=1}^N\beta _k|\varphi _N\otimes \downarrow \rangle \langle \varphi _k\otimes \uparrow | + \text{ h.c. } \end{aligned}$$

In other words,

7.3 $$\begin{aligned}&H_\alpha = |g\rangle \langle g|\otimes H_{a}^{(g)} + | e \rangle \langle e|\otimes H_{a}^{(e)}+ |g\rangle \langle e| \otimes |\varphi _1 \rangle \langle \Phi _\alpha | + |e\rangle \langle g|\otimes |\Phi _\alpha \rangle \langle \varphi _1 | \end{aligned}$$

7.4 $$\begin{aligned}&H_\beta = |\downarrow \rangle \langle \downarrow |\otimes H_{b}^{(\downarrow )} + | \uparrow \rangle \langle \uparrow |\otimes H_{b}^{(\uparrow )}+ |\downarrow \rangle \langle \uparrow | \otimes |\varphi _N \rangle \langle \Phi _\beta | + |\uparrow \rangle \langle \downarrow |\otimes |\Phi _\beta \rangle \langle \varphi _N | \end{aligned}$$

with $H_a^{(\#)}=\sum _{j=1}^N a_j^{(\#)}|\varphi _j\rangle \langle \varphi _j |$, $\#\in \{g,e\}$, $\Phi _\alpha = \sum _{k=1}^N\alpha _k\varphi _k$, and similarly for $H_\beta$, introducing $H_b^{(\#)}=\sum _{j=1}^N b_j^{(\#)}|\varphi _j\rangle \langle \varphi _j |$, $\#\in \{\downarrow , \uparrow \}$, and $\Phi _\beta = \sum _{k=1}^N\beta _k\varphi _k$.

On the one hand, this example shows our hypotheses can be checked for arbitrary *N* and, on the other hand, it can lead to physically relevant models under additional assumptions, see for instance Sect. 8 where we deal with qubits ($N=2$) subject to inter-qubit Coulomb interaction and flip-flop type interaction Hamiltonian.

With these definitions we compute

7.5 $$\begin{aligned} \overline{H}^{\, \tau }=&\, t_A H_{a}^{(g)}+ (1-t_A)H_{a}^{(e)}+ t_B H_{b}^{(\downarrow )}+ (1-t_B)H_{b}^{(\uparrow )} \nonumber \\ =&\sum _{j=1}^N \big (t_A a_j^{(g)}+ (1-t_A) a_j^{(e)} +t_B b_j^{(\downarrow )}+(1-t_B)b_j^{(\uparrow )} \big )|\varphi _j\rangle \langle \varphi _j |, \end{aligned}$$

which yields

7.6 $$\begin{aligned} \varphi _j^{\tau }=\varphi _j \ \ \text{ and } \ \ e_j^\tau =\big (t_A a_j^{(g)}+ (1-t_A) a_j^{(e)} +t_B b_j^{(\downarrow )}+(1-t_B)b_j^{(\uparrow )} \big ). \end{aligned}$$

We can choose the real parameters $a_j^{(g)}, a_j^{(e)}, b_j^{(\downarrow )}, b_j^{(\uparrow )}$ so that the generic assumption **Spec** $\overline{H}^{\, \tau }$ holds for any choice of $0<t_A, t_B <1$.

### Leading Order Term

The next step consists in determining the diagonal elements of the nonnegative operators *h*(*k*) defined in (6.11), $1\le k\le N$; more precisely $h_j(k):=\langle \varphi _j |h(k) \varphi _j\rangle$, for $j\ne k$. We first compute

7.7 $$\begin{aligned} \overline{H}^{\, \tau _A}&=H_\beta +(t_AH_a^{(g)}+(1-t_A)H_a^{(e)})\otimes {\mathbb {I}}_B \end{aligned}$$

7.8 $$\begin{aligned} \overline{H}^{\, \tau _B}&=H_\alpha +{\mathbb {I}}_A\otimes (t_BH_b^{(\downarrow )}+(1-t_B)H_b^{(\uparrow )}). \end{aligned}$$

Since we do not need the elements $\langle \varphi _k |h(k) \varphi _k\rangle$, we do not make explicit their contribution, that we generically denote below by $c_i(k) P_k$, where $c_i(k)\ge 0$, $i=1,2,3,4$. With this convention, we get for the different elements *h*(*k*) is made of

7.9 $$\begin{aligned} \mathrm{tr}_{AB}(H(\tau _A\otimes P_k^\tau \otimes \tau _B)H)= & {} c_1(k) P_k + (1-t_A)|\alpha _k|^2|\varphi _1\rangle \langle \varphi _1 |+\delta _{k,1}t_A|\Phi _\alpha \rangle \langle \Phi _\alpha | \nonumber \\&+ (1-t_B)|\beta _k|^2|\varphi _N\rangle \langle \varphi _N |+\delta _{k,N}t_B|\Phi _\beta \rangle \langle \Phi _\beta | \nonumber \\ \mathrm{tr}_{A}(\overline{H}^{\, \tau _B}(\tau _A\otimes P_k^\tau )\overline{H}^{\, \tau _B}))= & {} c_2(k) P_k+(1-t_A)|\alpha _k|^2|\varphi _1\rangle \langle \varphi _1 |+\delta _{k,1}t_A|\Phi _\alpha \rangle \langle \Phi _\alpha | \nonumber \\ \mathrm{tr}_{B}(\overline{H}^{\, \tau _A}( P_k^\tau \otimes \tau _B )\overline{H}^{\, \tau _A}))= & {} c_3(k) P_k+(1-t_B)|\beta _k|^2|\varphi _N\rangle \langle \varphi _N |+\delta _{k,N}t_B|\Phi _\beta \rangle \langle \Phi _\beta |.\nonumber \\ \end{aligned}$$

Eventually,

7.10 $$\begin{aligned} h(k)= & {} \frac{2}{\gamma _A \gamma _B}\Big \{ (1-t_A)|\alpha _k|^2 \gamma _B |\varphi _1\rangle \langle \varphi _1 | +\delta _{k,1}t_A \gamma _B |\Phi _\alpha \rangle \langle \Phi _\alpha |\nonumber \\&+\delta _{k,N} t_B \gamma _A |\Phi _\beta \rangle \langle \Phi _\beta |+(1-t_B) |\beta _k|^2 \gamma _A |\varphi _N\rangle \langle \varphi _N | \Big \} +c_4(k) P_k\,, \end{aligned}$$

The offdiagonal elements $h_j(k)$, $j\ne k$, of the matrix $-\Phi _D$ immediately follow: let7.11Therefore the matrix form (8.17) of the operator $\Phi _D$ reads7.12where the diagonal elements $\tilde{h}_j(j)=T_j+S_j$, $0<j<N$, $\tilde{h}_1(1)=\sum _{j=2}^{N} U_j+T_N$ and $\tilde{h}_N(N)=\sum _{j=2}^{N} V_j+S_N$.

Note that $\alpha _j\ne 0 \Leftrightarrow$ $S_j\ne 0$ and $U_j\ne 0$ , while $\beta _j\ne 0\Leftrightarrow$ $T_j\ne 0$ and $V_j\ne 0$. Hence, looking at the first row of (7.12), one sees that **Coup** holds for this model when

7.13 $$\begin{aligned} \alpha _2 \alpha _3 \dots \alpha _{N-1}\ne 0 \ \text{ and } \ |\beta _1|^2+|\alpha _N|^2\ne 0, \end{aligned}$$

or, looking at the last row, when

7.14 $$\begin{aligned} \beta _2\beta _3\dots \beta _{N-1}\ne 0\ \text{ and } \ |\beta _1|^2+|\alpha _N|^2\ne 0. \end{aligned}$$

In either cases, this validates the conclusions of Theorem 5.2 on the invariant state and the way to compute it. From now on, we assume that either (7.13) or (7.14) holds.

The leading term of the invariant state is determined by the one dimensional kernel of $\Phi _D$ which turns out to be computable explicitly. We have, noting that $S_j+T_j>0$ for $2\le j\le N-1$,

7.15 $$\begin{aligned} \mathrm{Ker}\,\Phi _D={\mathbb {C}}\begin{pmatrix} x_1 \\ x_2 \\ \vdots \\ x_N \end{pmatrix}, \ \ \text{ where } \ \ \left\{ \begin{matrix} x_1=S_N+\sum _{j=2}^{N-1}\frac{V_jS_j}{S_j+T_j}+V_1 \\ x_N=U_N+\sum _{j=2}^{N-1}\frac{U_jT_j}{S_j+T_j}+T_1 \\ x_j=\frac{U_jx_1+V_jx_N}{S_j+T_j}, \ 2\le j\le N-1\end{matrix}\right. . \end{aligned}$$

The corresponding faithful leading order $\rho _0$, *i.e.* $\rho _0>0$, of the invariant state of the QRM thus reads

7.16 $$\begin{aligned} \rho _0=\frac{1}{Z}\tau _A\otimes \sum _{j=1}^Nx_j |\varphi _j\rangle \langle \varphi _j| \otimes \tau _B, \ \ \text{ where } \ Z=\sum _{k=1}^Nx_k. \end{aligned}$$

Actually, the following more explicit expressions are true. With

7.17 $$\begin{aligned} y(N)=\sum _{j=2}^{N-1}\frac{\gamma _A\gamma _B|\alpha _j \beta _j|^2}{(1-t_A)\gamma _B|\alpha _j|^2+(1-t_B)\gamma _A|\beta _j|^2}, \end{aligned}$$

we can write

7.18 $$\begin{aligned} x_1&= (1-t_A)|\alpha _N|^2\gamma _B+y(N)t_B(1-t_A)+t_B|\beta _1|^2\gamma _A \end{aligned}$$

7.19 $$\begin{aligned} x_N&= t_A|\alpha _N|^2\gamma _B+y(N)t_A(1-t_B)+(1-t_B)|\beta _1|^2\gamma _A \end{aligned}$$

7.20 $$\begin{aligned} x_j&= t_A|\alpha _N|^2\gamma _B+t_B|\beta _1|^2\gamma _A+y(N)t_At_B \nonumber \\&\quad + \frac{\gamma _A\gamma _B (|\alpha _N|^2t_A(2t_B-1)|\beta _j|^2+|\beta _1|^2t_B(2t_A-1)|\alpha _j|^2)}{(1-t_A)\gamma _B|\alpha _j|^2+(1-t_B)\gamma _A|\beta _j|^2}, \end{aligned}$$

for $$2\le j \le N$$.

Note in particular the generic nontrivial dependence on *j* of the populations of (the reduced) leading order $\rho _0$ of the invariant state. Further remarks are in order:

- For non zero coefficients $\alpha _j$ and $\beta _j$, $x_j$ is independent of $2\le j\le N-1$ if
  7.21 $$\begin{aligned} \frac{(2t_A-1)t_B}{(1-t_A)}\gamma _A|\beta _1|^2=\frac{(2t_B-1)t_A}{(1-t_B)}\gamma _B|\alpha _N|^2. \end{aligned}$$
- In case we consider thermal states for $\tau _\#$ on ${\mathbb {C}}^2$, $\#\in \{A,B\}$, such that $t_\#=\frac{1}{1+e^{-\beta _\#E_\#}}$, with excitation energy $E_\#>0$. We get that $t_\#\rightarrow 1$ when $\beta _\#\rightarrow \infty$, while $t_\#\rightarrow 1/2$ when $\beta _\#\rightarrow 0$, which shows that at high temperature, the populations tend to be constant.

## Example on ${\mathbb {C}}^2\otimes {\mathbb {C}}^2\otimes {\mathbb {C}}^2$

With the previous example considering $H_C \in {\mathbb {C}}^N$, we could derive the exact expressions of the map $\Phi _D$ and of the leading order solution. However, going to first order correction and beyond requires considerable effort and would not be enlightening for the reader. This motivates this second example, where we restrict $H_C$ to be in ${\mathbb {C}}^2$ and consider an interaction Hamiltonian *H* that is appropriate to describe effective physical systems. The goal of this section is twofold. First, we derive explicitly higher order corrections illustrating the theorems of Sect. 5, showing that we can capture the main features of the dynamics with relatively little effort as compared to the complexity of the system. Second, we make a clear connection between a tri-partite quantum reset model and models suitable to describe realistic physical systems.

### Model

Explicitly, we consider here a chain of three qubits characterized by their bare energies $e_A, e_B, e_C$ entering $H_0$. They are interacting through *H*. The two Hamiltonians are given by

8.1 $$\begin{aligned} H_0= & {} e_A |1\rangle \langle 1 | \otimes {\mathbb {I}}_C \otimes {\mathbb {I}}_B + {\mathbb {I}}_A \otimes e_C |1\rangle \langle 1 | \otimes {\mathbb {I}}_B + {\mathbb {I}}_A \otimes {\mathbb {I}}_C \otimes e_B |1\rangle \langle 1 |\,, \end{aligned}$$

8.2 $$\begin{aligned} H= & {} U\, (|11\rangle _{AC} \langle 11 | \otimes {\mathbb {I}}_B + {\mathbb {I}}_A \otimes (|11\rangle _{CB} \langle 11 | ) \nonumber \\&+ ( J_\alpha |01\rangle _{AC} \langle 10 | \otimes {\mathbb {I}}_B + {\mathbb {I}}_A \otimes J_\beta |01\rangle _{CB} \langle 10 | + h.c)\,. \end{aligned}$$

Without loss of generality, we assume the interaction strengths $U,J_\alpha , J_\beta$ to be real. This model could be effective for instance for three qubits subject to nearest-neighbour interactions: a Coulomb interaction (set by *U*) whenever two adjacent qubits are occupied and to a flip-flop interaction term of the form $\vert 01 \rangle \langle 10 \vert + h.c.$ that conserves the number of excitations (set by $J_\alpha , J_\beta$ with $J_\alpha \ne J_\beta$). In the ordered computational basis of the three qubits

8.3 $$\begin{aligned} \{ |000\rangle , |001\rangle , |010\rangle , |011\rangle , |100\rangle , |101\rangle , |110\rangle , |111\rangle \}, \end{aligned}$$

the total Hamiltonian $H_{tot} = H_0 + g H$ reads

8.4 $$\begin{aligned}&H_{tot} = \nonumber \\&\quad \left( \begin{array}{cccccccc} 0 &{} 0&{} 0 &{} 0 &{} 0 &{} 0 &{} 0 &{} 0 \\ 0 &{} e_B &{} g J_\beta &{} 0 &{} 0 &{}0 &{} 0 &{} 0 \\ 0 &{} g J_\beta &{} e_C &{} 0 &{} g J_\alpha &{}0 &{} 0 &{} 0 \\ 0 &{} 0 &{} 0 &{} e_B + e_C +g U &{} 0 &{}g J_\alpha &{} 0 &{} 0 \\ 0 &{} 0 &{}g J_\alpha &{} 0 &{} e_A &{}0 &{} 0 &{} 0 \\ 0 &{} 0 &{} 0 &{} g J_\alpha &{} 0 &{} e_A+e_B &{}g J_\beta &{} 0 \\ 0 &{} 0 &{} 0 &{} 0 &{} 0 &{} g J_\beta &{} e_A + e_C + g U &{} 0 \\ 0 &{} 0 &{} 0 &{} 0 &{} 0 &{}0 &{} 0 &{} e_A + e_B + e_C + 2 g U \\ \end{array}\right) . \end{aligned}$$

This model corresponds exactly to the previous example with $N=2$ and setting:

8.5 $$\begin{aligned}&\alpha _1=\beta _2=a_2^{(e)} =a_1^{(g)} =a_2^{(g)} =b_2^{(\downarrow )}= {b_2^{(\uparrow )}} =b_1^{(\uparrow )} = 0 \end{aligned}$$

8.6 $$\begin{aligned}&a_1^{(e)} = b_1^{(\downarrow )} = U, \ \alpha _2=J_\alpha , \ \beta _1=J_\beta , \end{aligned}$$

The ground state for the three qubits is now simply given by $\vert 000 \rangle$ and corresponds to $\vert g \otimes \varphi _2 \otimes \uparrow \rangle$ in the previous example with $N=2$. For clarity, we provide the expression of $H_{tot}$ in the form introduced in (7.2)

8.7 $$\begin{aligned} H_\alpha&= U |e\otimes { \varphi _1}\rangle \langle e\otimes {\varphi _1} | +J_\alpha | {g}\otimes \varphi _1 \rangle \langle {e}\otimes \varphi _2 | + \text{ h.c. }\nonumber \\ H_\beta&= U |{ \varphi _1 }\otimes {\downarrow } \rangle \langle {\varphi _1}\otimes {\downarrow } |+ J_\beta | \varphi _2\otimes \downarrow \rangle \langle \varphi _1\otimes \uparrow | + \text{ h.c. } \end{aligned}$$

The two ends (*A* and *B*) of the 3-qubit chain are weakly coupled to their own thermal baths at inverse temperatures $\beta _A$ and $\beta _B$ with coupling strengths $\gamma _A$ and $\gamma _B$ respectively. Dissipation takes place following QRM . The reset states are assumed to be thermal states defined by the Maxwell-Boltzmann distribution with their respective inverse temperature $\beta _\# = 1/T_\#$ ($k_B = 1$ in the following) in the basis $\{ \vert 0 \rangle , \vert 1 \rangle \}$:

8.8 $$\begin{aligned} \tau _\#=\frac{1}{Z_\#}\left( \begin{array}{cc} 1&{}0 \\ 0 &{} e^{-\beta _\#e_\#} \end{array}\right) =\left( \begin{array}{cc} t_\#&{}0 \\ 0 &{} 1-t_\# \end{array}\right) , \ \ \#\in \{A, B\}. \end{aligned}$$

Note that since the ground state $\vert 0 \rangle$ in the *C* part of the Hilbert space corresponds to $|\uparrow \rangle$, the substitution $t_B\rightarrow (1-t_B)$ is in order to use the results of Sect. 7.

Let us remark that this model for a tri-partite open quantum system differs from previous works on reset models in the context of quantum thermodynamics, studying in particular quantum absorption refrigerators and entanglement engines, Refs. [4, 30, 32]. These models consist of a chain of 2, 3 or N qubits, each of them being coupled to its own thermal bath. Dissipation due to the presence of environments is captured through QRM . In Ref. [30], the steady-state solution for 3 qubits with three different environments is derived analytically, whereas the case of two qubits is fully solved in Ref. [4]. In contrast, in this work, we derive the steady-state solution considering an arbitrary system *C* only coupled to the two ends *A* and *B* of the chain, as long as $H_C$ satisfies generic assumptions.

### Generic Assumptions

We first check the assumptions for $H_C$ and *H*. The condition $\mathbf{Spec}(H_C)$, is trivially satisfied in this case as the spectrum $\sigma (H_C) = \{ 0, e_C\}$ is simple with $e_C \ne 0$. We can then verify $\mathbf{Spec}(\overline{H}^\tau )$ with

8.9 $$\begin{aligned} \overline{H}^\tau = \mathrm{tr}_{AB}( H \tau _A \otimes {\mathbb {I}}_C \otimes \tau _B)=U(2-t_A-t_B) |1\rangle \langle 1 |\,, \end{aligned}$$

as defined by Eq. (4.13) . The spectrum $\sigma (\bar{H}^\tau ) = \{ 0, U ( 2 - t_A - t_B)\}$ with associated eigenvectors $\{|0\rangle , |1\rangle \}$ is simple whenever $U \ne 0$ and $t_A + t_B \ne 2$ where $t_A, t_B$ stand for the ground state populations of the reset states $\tau _A, \tau _B$. The identity $t_A + t_B = 2$ is only satisfied for zero temperature reservoirs, $t_A = t_B = 1$. Hence we stay in the generic case, $t_A, t_B < 1$. The condition $U \ne 0$ also tells us that a flip-flop interaction Hamiltonian of the form $(\vert 0 1 \rangle \langle 1 0 \vert + h.c.)$ is not sufficient to ensure the required non-degeneracy conditions in the 0-subspace of $\mathcal{L}_0$. We easily verify that the kernel of $\mathcal{L}_0$ has dimension $n_C^2 = 4$ if $H_C = 0$ and $n_C = 2$ if $H_C \ne 0$.

In the following, we will restrict the derivation of the steady-state solution up to the second order correction assuming no drive, *i.e.* $H_A = H_B = H_C = 0$. Let us note that in two dimensions, there is no loss of generality to consider the reset states $\tau _A$ and $\tau _B$ defined as thermal states with respect to $H_A$ and $H_B$.

### Leading Order Solution, No Drive

Under **Spec**($\bar{H}^\tau$) and Lemma 4.1, the first-order-correction projector $\tilde{Q}_0 \tilde{\mathcal{L}}_1 \tilde{Q}_0$ in the 0-eigenvalue subspace is fully characterized by the map $\Phi$ acting onto $\mathcal{H}_C$, see Eq. (4.25) and Theorem 4.3

8.10 $$\begin{aligned} \Phi (\cdot ):= {\mathrm{tr}}_{AB}\big (\big [H, \mathcal{L}_0^{-1}([H, \tau _A\otimes \mathrm{Diag}(\, \cdot \,) \otimes \tau _B])\big ]\big ) : \mathcal{B}(\mathcal{H}_C)\rightarrow \mathcal{B}(\mathcal{H}_C)\cap \{\rho _C\, | \, \mathrm{tr}\rho _C=~0\}. \end{aligned}$$

In contrast to the previous example, we can compute explicitly here the map $\Phi$ and not only $\Phi _D$. To this end, we consider $\rho _C$ to be initially in an arbitrary diagonal state (with respect to the eigenbasis of $\overline{H}^\tau$)

8.11 $$\begin{aligned} \rho _C = \left( \begin{array}{cc} r_C^0 &{} 0 \\ 0 &{} r_C^1 \end{array} \right) \,. \end{aligned}$$

Defining the linear form on ${\mathbb {C}}^2$

8.12 $$\begin{aligned} X(r_C^0,r_C^1) =- r_C^0 (J_\beta ^2 (1-t_B) \gamma _A + J_\alpha ^2 (1-t_A) \gamma _B)+ r_C^1 (J_\beta ^2 t_B \gamma _A + J_\alpha ^2 t_A \gamma _B) \,, \end{aligned}$$

we find the matrix $\Phi (\rho _C) \in \mathcal{B}(\mathcal{H}_C)$ to be given by (with respect to the eigenbasis of $\overline{H}^\tau$)

8.13 $$\begin{aligned} \Phi (\rho _C) = \frac{2}{\gamma _A \gamma _B} \left( \begin{array}{cc} X(r_C^0,r_C^1) &{} 0 \\ 0 &{}- X(r_C^0,r_C^1) \end{array}\right) \,. \end{aligned}$$

Note that $\Phi (\rho _C)$ is diagonal, so that for this example we have $\Phi (\cdot )=\Phi _D(\cdot )$. In particular

8.14 $$\begin{aligned} \mathrm{Ker}\,\Phi _D(\cdot )={\mathbb {C}}\left( \begin{array}{cc} \gamma _A t_B J_\beta ^2 + \gamma _B t_A J_\alpha ^2 &{} 0 \\ 0 &{} \gamma _A (1-t_B) J_\beta ^2 + \gamma _B (1-t_A) J_\alpha ^2 \end{array} \right) \end{aligned}$$

is one dimensional, so that Assumption **Coup** is satisfied. Then $\mathrm{Ker}\,\Phi _D$ provides the leading order steady-state solution $\rho _{0} = \tau _A \otimes \rho _C^{(0)} \otimes \tau _B$ with

8.15 $$\begin{aligned} \rho _C^{(0)}= \left( \begin{array}{cc} \frac{ \gamma _A t_B J_\beta ^2+ \gamma _B t_A J_\alpha ^2}{\gamma _A J_\beta ^2+ \gamma _B J_\alpha ^2} &{} 0 \\ 0 &{} \frac{\gamma _A (1-t_B) J_\beta ^2+ \gamma _B (1-t_A) J_\alpha ^2}{\gamma _A J_\beta ^2+ \gamma _B J_\alpha ^2} \end{array} \right) \,. \end{aligned}$$

Interestingly, the zeroth order solution is the exact solution in the equilibrium situation, i.e. when $\tau _A = \tau _B = \tau$, the state $\rho _0 = \ \tau \otimes \tau \otimes \tau$ satisfies for any $g\in {\mathbb {R}}$ (or ${\mathbb {C}}$) $\mathcal{L}_g (\rho _0) = 0,$ an instance of Remark ii) 5.3.

#### Remark 8.1

In this example, the matrix $\Phi _D$ can also be derived directly from the previous example with $N=2$, starting from the positive operator *h*(*k*):

8.16 $$\begin{aligned} h(k)= & {} \frac{2}{\gamma _A \gamma _B} \Big (\vert \alpha _k \vert ^2 (1- t_A) \gamma _B \vert 1\rangle \langle 1 \vert + \vert \beta _k \vert ^2 t_B \gamma _A \vert 0 \rangle \langle 0 \vert \nonumber \\&\, + t_A \gamma _B \vert 0 \rangle \langle 0 \vert + (1-t_B) \gamma _A \vert 1\rangle \langle 1 \vert \,\, \Big ) + c_4(k) P(k)\,. \end{aligned}$$

In the basis $\{ \vert 0 \rangle \langle 0|, \vert 1 \rangle \langle 1|\}$, given (8.5), the substitution $t_B\rightarrow 1-t_B$, and according to (7.12), the matrix $\Phi _D$ reads

8.17 $$\begin{aligned} \Phi _D= \frac{-2}{\gamma _A \gamma _B} \left( \begin{array}{cc} \gamma _A J_\beta ^2 (1-t_B) + \gamma _B J_\alpha ^2 (1-t_A) &{} - \gamma _A J_\beta ^2 t_B - \gamma _B J_\alpha ^2t_A \\ -\gamma _A J_\beta ^2 (1-t_B) - \gamma _B J_\alpha ^2 (1-t_A) &{} \gamma _A J_\beta ^2 t_B + \gamma _B J_\alpha ^2 t_A \end{array}\right) \,, \end{aligned}$$

whose kernel in this same basis is generated by the two-dimensional vector

8.18 $$\begin{aligned} \mathrm{Ker}\,\Phi _D = {\mathbb {C}}(\gamma _A J_\beta ^2 t_B + \gamma _B J_\alpha ^2 t_A, \gamma _A J_\beta ^2 (1-t_B) + \gamma _B J_\alpha ^2 (1-t_A))^T\,. \end{aligned}$$

Let us note that $\Phi _D$, when written as a superoperator acting onto diagonal matrices, takes a diagonal form, see Eq. (8.13).

### Underlying Markov Process

We have enough information here to determine the natural two-state classical continuous Markov process associated to the model, according to Theorem 6.5. The state space is denoted by $\{0,1\}\equiv \{| 0 \rangle \langle 0 |, | 1 \rangle \langle 1 |\}$, and by (6.24) we need to compute $e^{s \Phi _D}$ to determine the transition probabilities of the process

8.19 $$\begin{aligned} {\mathbb {P}}(X_s=j|X_0=k)=\mathrm{tr}\big \{ |k\rangle \langle k | e^{s \Phi _D}(| j \rangle \langle j |)\big \}\equiv (e^{s\Phi _D})_{k,j} , \ \ 0\le j,k\le 1 \end{aligned}$$

The spectral decomposition of $\Phi _D$ in the matrix form (8.17) is easily obtained. Introducing

8.20 $$\begin{aligned} \varphi _+=\gamma _A J_\beta ^2 t_B + \gamma _B J_\alpha ^2 t_A, \ \ \varphi _-=\gamma _A J_\beta ^2 (1-t_B) + \gamma _B J_\alpha ^2 (1-t_A), \end{aligned}$$

we have

8.21 $$\begin{aligned} \sigma (\Phi _D)=\{0, -2(\varphi _++\varphi _-)/(\gamma _A \gamma _B)\}=\{0, -2(\gamma _A J_\beta ^2 + \gamma _B J_\alpha ^2 )/(\gamma _A \gamma _B)\}, \end{aligned}$$

with eigenvector associated to the non zero eigenvalue proportional to $$\begin{pmatrix}1&-1 \end{pmatrix}^T$$. Hence,

8.22 $$\begin{aligned} \Phi _D=\frac{-2(\varphi _++\varphi _-)}{\gamma _A \gamma _B}Q_+ + 0\, Q_0, \end{aligned}$$

with spectral projectors

8.23 $$\begin{aligned} Q_0=\frac{\Big | \begin{pmatrix}\varphi _+ \\ \varphi _- \end{pmatrix}\Big \rangle \Big \langle \begin{pmatrix}1 \\ 1 \end{pmatrix} \Big |}{\varphi _-+\varphi _+} , \ \ Q_+=\frac{\Big | \begin{pmatrix}1 \\ -1 \end{pmatrix}\Big \rangle \Big \langle \begin{pmatrix}\varphi _- \\ -\varphi _+ \end{pmatrix} \Big |}{\varphi _-+\varphi _+}. \end{aligned}$$

Therefore, with $$\tilde{s}=\frac{2s(\varphi _++\varphi _-)}{\gamma _A \gamma _B}$$,

8.24 $$\begin{aligned} e^{s \Phi _D}=e^{-\tilde{s}}Q_+ + Q_0=\frac{1}{\varphi _-+\varphi _+} \begin{pmatrix} \varphi _+ + e^{-\tilde{s}}\varphi _- &{} \varphi _+ - e^{-\tilde{s}}\varphi _+\\ \varphi _- - e^{-\tilde{s}}\varphi _- &{} \varphi _- + e^{-\tilde{s}}\varphi _+\end{pmatrix} . \end{aligned}$$

In turn this eventually yields the sought for transition probabilities

8.25 $$\begin{aligned} {\mathbb {P}}(X_s=0|X_0=0)&=\frac{\varphi _+ + e^{-\tilde{s}}\varphi _-}{\varphi _-+\varphi _+}, \ \ {\mathbb {P}}(X_s=1|X_0=1)=\frac{\varphi _- + e^{-\tilde{s}}\varphi _+}{\varphi _-+\varphi _+},\nonumber \\ {\mathbb {P}}(X_s=1|X_0=0)&=\frac{\varphi _-(1 - e^{-\tilde{s}})}{\varphi _-+\varphi _+}, \ \ {\mathbb {P}}(X_s=0|X_0=1)=\frac{\varphi _+(1 - e^{-\tilde{s}})}{\varphi _-+\varphi _+}. \end{aligned}$$

We stress that in absence of leading order driving Hamiltonian, the state space of the Markov process into play is determined by the eigenstates of $\overline{H}^\tau$, that takes into account the effects of the reset matrices.

### Higer-Order Corrections, No Drive

We now illustrate Theorem 5.2 by deriving the converging expansion of the unique invariant state of $\mathcal{L}_g$

8.26 $$\begin{aligned} \rho _0(g) = \rho _0 + g \, \rho _1 + g^2 \, \rho _2 + \ldots \, \ \text{ with }, \ \ \rho _0 = \tau _A \otimes \rho _C^{(0)} \otimes \tau _B\,, \end{aligned}$$

and

8.27 $$\begin{aligned} \rho _j = R_j + \tau _A \otimes r_C^{(j)} \otimes \tau _B \quad \forall j \ge 1\,. \end{aligned}$$

We recall the definitions for convenience

$$\begin{aligned} R_j= & {} i\mathcal{L}_0^{-1}([H, \rho _{j-1}]),\\ \mathrm{Offdiag}_\tau r_C^{(j)}= & {} -i[\overline{H}^{\, \tau }, \cdot ]^{-1}\Big ( \mathrm{Offdiag}_\tau \mathrm{tr}_{AB}\big (\big [H, \mathcal{L}_0^{-1}([H, \rho _{j-1}])\big ]\big )\Big ),\\ \mathrm{Diag}_\tau r_C^{(j)}= & {} -\Phi _D^{-1}\big (\mathrm{Diag}_\tau \mathrm{tr}_{AB}([H,\mathcal{L}_0^{-1}(i[H, R_{j}+\tau _A\otimes \mathrm{Offdiag}_\tau r_C^{(j)}\otimes \tau _B])])\big )\,. \end{aligned}$$

For the first-order correction, we start computing $R_1=i\mathcal{L}_0^{-1}([H, \tau _A \otimes \rho _C^{(0)} \otimes \tau _B])$ which can be expressed with $F=i(|01\rangle \langle 10 | -|10\rangle \langle 01 | )$ (acting on $\mathcal{H}_A\otimes \mathcal{H}_C$ or $\mathcal{H}_C\otimes \mathcal{H}_B$ depending on the context) as

8.28 $$\begin{aligned} R_1 =&\frac{(t_A-t_B)J_\alpha J_\beta }{\gamma _A J_\beta ^2+ \gamma _B J_\alpha ^2}\Big (J_\beta F\otimes \tau _B+ J_\alpha \tau _A\otimes F \Big ) \nonumber \\ =&\frac{(t_A-t_B)J_\alpha J_\beta }{\gamma _A J_\beta ^2+ \gamma _B J_\alpha ^2}\Big ( J_\beta \, i(|01\rangle \langle 10 | -|10\rangle \langle 01 | )\otimes \tau _B+ J_\alpha \tau _A\otimes \, i(|01\rangle \langle 10 | -|10\rangle \langle 01 | ) \Big ). \end{aligned}$$

We first note that since $\Phi =\Phi _D$, the expression for $\mathrm{Offdiag}_\tau r_C^{(1)}$ reduces to zero:

8.29 $$\begin{aligned} \mathrm{Offdiag}_\tau r_C^{(1)}&=-i[\overline{H}^{\, \tau }, \cdot ]^{-1}\Big ( \mathrm{Offdiag}_\tau \Phi (\rho _C^{(0)})\Big )\equiv 0. \end{aligned}$$

Then, it remains to determine $\mathrm{Diag}_\tau r_C^{(1)}$ to get the first order correction in *g*. Thanks to (8.29) and using (8.28) for $R_1$, we compute

8.30 $$\begin{aligned} \mathrm{Diag}_\tau r_C^{(1)}=-\Phi _D^{-1}\big (\mathrm{Diag}_\tau \mathrm{tr}_{AB}([H,\mathcal{L}_0^{-1}(i[H, R_{1}])])\big )=0. \end{aligned}$$

Hence, the first order correction is simply given by $R_1$, $\rho _1 = R_1$ and we obtain

8.31 $$\begin{aligned} \rho _0(g) = \tau _A \otimes \rho _C^{(0)}\otimes \tau _B + g R_1+ \mathcal{O}(g^2)\,. \end{aligned}$$

We proceed with the second-order correction and compute $R_2 = i \mathcal{L}_0^{-1} \big ( [H, R_1] \big )$. The matrix $R_2$ is rather complex and we provide the expressions for its diagonal and off-diagonal elements separately. Its 8 diagonal elements in the ordered basis (8.3) are proportional to by

8.32 $$\begin{aligned} \mathrm{Diag}(R_2)= & {} \frac{J_\alpha J_\beta }{\gamma _A \gamma _B} \Bigg ( t_A t_B (\gamma _A - \gamma _B), - t_A (t_B \gamma _A + \gamma _B (1-t_B)), \nonumber \\&\quad t_A \gamma _A (1-t_B) - t_B \gamma _B (1 -t_A), (1-t_B) (t_A \gamma _A - \gamma _B (1-t_A)), \nonumber \\&\quad t_B (\gamma _B t_A + \gamma _A (1-t_A)), \gamma _A t_A (1-t_B) - \gamma _A t_B (1-t_A),\nonumber \\&\quad (1-t_A) (\gamma _B t_B + \gamma _A (1-t_B)), (1-t_A) (1-t_B) (\gamma _B - \gamma _A)\, \Bigg ). \end{aligned}$$

For its off-diagonal elements, we introduce $F_2 = |01\rangle \langle 10 | + |10\rangle \langle 01 \vert$ and the coefficient matrices

8.33 $$\begin{aligned} \Gamma _A = \left( \begin{array}{cc} \gamma _A &{} 0 \\ 0 &{} \gamma _A + \gamma _B/(1-t_A) \end{array} \right) \quad ; \quad \Gamma _B = \left( \begin{array}{cc} \gamma _B &{} 0 \\ 0 &{} \gamma _B + \gamma _A/(1-t_B) \end{array} \right) \,. \end{aligned}$$

The matrix $R_2$ can then be written in a compact form

8.34 $$\begin{aligned} R_2= & {} \frac{2 J_\alpha J_\beta (t_A - t_B)}{J_\beta ^2 \gamma _A + J_\alpha ^2 \gamma _B} \Big \{ \mathrm{Diag}(R_2) + \frac{1}{\gamma _A + \gamma _B}\nonumber \\&\times \Big ( \frac{- J_\alpha U (1-t_A)}{2 \gamma _B} \tau _A \Gamma _A \otimes F_2 + \frac{J_\beta U (1-t_B)}{2 \gamma _A} F_2 \otimes \tau _B \Gamma _B \nonumber \\&- \frac{1}{2} (J_\alpha ^2 t_A - J_\beta ^2 t_B) \vert 001 \rangle \langle 100 \vert + \frac{1}{2} (J_\alpha ^2 (1-t_A ) - J_\beta ^2 (1-t_B)) \vert 110 \rangle \langle 011 \vert \Big ) \Big \}.\nonumber \\ \end{aligned}$$

For $\mathrm{Offdiag}r_C^{(2)}$, we find that it is equal to 0. This leads us to:

8.35 $$\begin{aligned} \mathrm{Diag}\, r_C^{(2)} = \left( \begin{array}{cc} X^{(2)}&{} 0 \\ 0 &{} - X^{(2)} \end{array} \right) \end{aligned}$$

with

8.36 $$\begin{aligned} X^{(2)}= & {} \frac{2 i J_\alpha ^2 J_\beta ^2 (t_A - t_B)}{ \gamma _A^2 \gamma _B^2 (\gamma _A + \gamma _B) (J_\beta ^2 \gamma _A + J_\alpha ^2 \gamma _B)} \nonumber \\&\times \Big \{ (\gamma _A + \gamma _B) ( J_\beta ^2 \gamma _A (2 \gamma _A - \gamma _B) - J_\alpha ^2 \gamma _B (2 \gamma _B - \gamma _A) )\nonumber \\&+ U^2 ( (1-t_A) \gamma _A^2( \gamma _B + (1-t_A) \gamma _A) - (1-t_B) \gamma _B^2 (\gamma _A - (1-t_B) \gamma _B)) \Big \}\,. \end{aligned}$$

The solution up to the second-order correction is then given by

8.37 $$\begin{aligned} \rho _0(g) = \tau _A \otimes ( \rho _C^{(0)} + g^2 r_C^{(2)}) \otimes \tau _B + g \, R_1 + g^2 R_2 + \mathcal{O}(g^3)\,. \end{aligned}$$

We note that coulomb-interaction term like in *U* starts playing a role when considering the second-order correction.

## Appendix

We provide here the proof of Proposition 6.7.

### Proof

By computations similar to those performed in the determination of $\Phi _D$, we have with $P_{jk}^\tau =|\varphi _j^\tau \rangle \langle \varphi _k^\tau |$,

9.1 $$\begin{aligned}&[H,\mathcal{L}_0^{-1}( [H,\tau _A\otimes P_{jk}^\tau \otimes \tau _B])]=-\frac{1}{\gamma _A+\gamma _B} [H,[H,\tau _A\otimes P_{jk}^\tau \otimes \tau _B]]\nonumber \\&\quad -\frac{\gamma _B/\gamma _A}{\gamma _A+\gamma _B}[H,[\overline{H}^{\, \tau _B},\tau _A\otimes P_{jk}^\tau ]\otimes \tau _B]-\frac{\gamma _A/\gamma _B}{\gamma _A+\gamma _B}[H,\tau _A\otimes [\overline{H}^{\, \tau _A}, P_{jk}^\tau \otimes \tau _B]]\nonumber \\&\quad -\frac{\gamma _A^2+\gamma _A\gamma _B+\gamma _B^2}{\gamma _A\gamma _B(\gamma _A+\gamma _B)}(e_j^\tau -e_k^\tau )[H,\tau _A\otimes P_{jk}^\tau \otimes \tau _B]. \end{aligned}$$

The last term in (9.1) yields the following contribution to $\tilde{\lambda }_{jk}^{(1)}$, using cyclicity of the trace and $\overline{H}^{\, \tau }\varphi _j^\tau =e_j^\tau \varphi _j^\tau$,

9.2 $$\begin{aligned} -\frac{\gamma _A^2+\gamma _A\gamma _B+\gamma _B^2}{\gamma _A\gamma _B(\gamma _A+\gamma _B)} (e_j^\tau -e_k^\tau )^2<0. \end{aligned}$$

Then, using cyclicity of the trace and $P_{kj}^\tau P_{jk}^\tau =P_{kk}^\tau$, we have

9.3 $$\begin{aligned}&\mathrm{tr}({\mathbb {I}}_A\otimes P_{kj}^\tau \otimes {\mathbb {I}}_B[H,[H,\tau _A\otimes P_{jk}^\tau \otimes \tau _B]])=-2\mathrm{tr}({\mathbb {I}}_A\otimes P_{kj}^\tau \otimes {\mathbb {I}}_B H \tau _A\otimes P_{jk}^\tau \otimes \tau _B H) \nonumber \\&\qquad +\mathrm{tr}(H(\tau _A\otimes P_{jj}^\tau \otimes \tau _B)H)+\mathrm{tr}(H(\tau _A\otimes P_{kk}^\tau \otimes \tau _B)H) \nonumber \\&\quad =\mathrm{tr}(H(\tau _A\otimes P_{jj}^\tau \otimes \tau _B)H)+\mathrm{tr}(H(\tau _A\otimes P_{kk}^\tau \otimes \tau _B)H)\nonumber \\&\qquad -2(\mathrm{tr}_{AB}(H (\tau _A\otimes P_{jk}^\tau \otimes \tau _B) H))_{jk}. \end{aligned}$$

Here $A_{jk}$ denotes the *jk* of the matrix $A\in \mathcal{B}(\mathcal{H}_C)$ with respect to the basis $\{\varphi _j^\tau \}$. Note that the operators in the full traces are non negative, whereas the last term is *a priori* complex valued.

Similarly,

9.4 $$\begin{aligned}&\mathrm{tr}({\mathbb {I}}_A\otimes P_{kj}^\tau \otimes {\mathbb {I}}_B [H,\tau _A\otimes [\overline{H}^{\, \tau _A}, P_{jk}^\tau \otimes \tau _B]])=\nonumber \\&\quad \mathrm{tr}_{AC} (\overline{H}^{\, \tau _A}(P_{jj}^\tau \otimes \tau _B)\overline{H}^{\, \tau _A})+\mathrm{tr}_{AC} (\overline{H}^{\, \tau _A}(P_{kk}^\tau \otimes \tau _B)\overline{H}^{\, \tau _A}) -2(\mathrm{tr}_{B}(\overline{H}^{\, \tau _A} P_{jk}^\tau \otimes \tau _B \overline{H}^{\, \tau _A}))_{jk}, \end{aligned}$$

and the analogous formula holds for the term involving $\overline{H}^{\, \tau _B}$. These expressions allow us to bound below their real part by a non negative quantity, as the next lemma shows. $\square$

### Lemma 9.1

Under the hypotheses above, we compute

9.5 $$\begin{aligned}&\mathfrak {R}\,\mathrm{tr}\Big \{({\mathbb {I}}_A\otimes P_{kj}^\tau \otimes {\mathbb {I}}_B)\big [H,[H,\tau _A\otimes P_{jk}^\tau \otimes \tau _B]\big ]\Big \} \nonumber \\&\quad \ge \mathrm{tr}\Big \{({\mathbb {I}}_A\otimes ({\mathbb {I}}_C- P_{jj}^\tau )\otimes {\mathbb {I}}_B)H(\tau _A\otimes P_{jj}^\tau \otimes \tau _B)H({\mathbb {I}}_A\otimes ({\mathbb {I}}_C-P_{jj}^\tau )\otimes {\mathbb {I}}_B) \Big \} \nonumber \\&\quad + \text{ same } \text{ with } \ k\leftrightarrow j. \end{aligned}$$

9.6 $$\begin{aligned}&\mathfrak {R}\,\mathrm{tr}\Big \{{\mathbb {I}}_A\otimes P_{kj}^\tau \otimes {\mathbb {I}}_B [H,\tau _A\otimes [\overline{H}^{\, \tau _A}, P_{jk}^\tau \otimes \tau _B]]\Big \} \nonumber \\&\quad \ge \mathrm{tr}\Big \{(({\mathbb {I}}_C-P_{jj}^\tau )\otimes {\mathbb {I}}_B) \overline{H}^{\, \tau _A}(P_{jj}^\tau \otimes \tau _B)\overline{H}^{\, \tau _A}(({\mathbb {I}}_C-P_{jj}^\tau )\otimes {\mathbb {I}}_B) \Big \} \nonumber \\&\quad + \text{ same } \text{ with } \ k\leftrightarrow j. \end{aligned}$$

9.7 $$\begin{aligned}&\mathfrak {R}\,\mathrm{tr}\Big \{{\mathbb {I}}_A\otimes P_{kj}^\tau \otimes {\mathbb {I}}_B)\big [H,[\overline{H}^{\, \tau _B},\tau _A\otimes P_{jk}^\tau ]\otimes \tau _B\big ]\Big \} \nonumber \\&\quad \ge \mathrm{tr}\Big \{({\mathbb {I}}_A\otimes ({\mathbb {I}}_C-P_{jj}^\tau ))\overline{H}^{\, \tau _B}(\tau _A\otimes P_{jj}^\tau )\overline{H}^{\, \tau _B}({\mathbb {I}}_A\otimes ({\mathbb {I}}_C-P_{jj}^\tau )) \Big \} \nonumber \\&\quad + \text{ same } \text{ with } \ k\leftrightarrow j. \end{aligned}$$

### Remark 9.2

Since

9.8 $$\begin{aligned}&({\mathbb {I}}_A\otimes ({\mathbb {I}}_C- P_{jj}^\tau )\otimes {\mathbb {I}}_B)H(\tau _A\otimes P_{jj}^\tau \otimes \tau _B)H({\mathbb {I}}_A\otimes ({\mathbb {I}}_C-P_{jj}^\tau )\otimes {\mathbb {I}}_B)=\nonumber \\&\quad ((\tau _A^{1/2}\otimes P_{jj}^\tau \otimes \tau _B^{1/2})H({\mathbb {I}}_A\otimes ({\mathbb {I}}_C-P_{jj}^\tau )\otimes {\mathbb {I}}_B))^*\nonumber \\&\quad \times ((\tau _A^{1/2}\otimes P_{jj}^\tau \otimes \tau _B^{1/2})H({\mathbb {I}}_A\otimes ({\mathbb {I}}_C-P_{jj}^\tau )\otimes {\mathbb {I}}_B)) \end{aligned}$$

is a non negative operator, we get from (9.1), (9.2) and the Lemma that

9.9 $$\begin{aligned} \mathfrak {R}\tilde{\lambda }_{jk}^{(1)}\le -\frac{\gamma _A^2+\gamma _A\gamma _B+\gamma _B^2}{\gamma _A\gamma _B(\gamma _A+\gamma _B)}(e_j^\tau -e_k^\tau )^2<0, \end{aligned}$$

which proves Proposition 6.7.

### Proof

We prove the first inequality, the others are similar. Let $G=H(\tau _A^{1/2}\otimes {\mathbb {I}}_C \otimes \tau _B^{1/2})$, so that the real part we need to consider reads, see (9.3),

9.10 $$\begin{aligned} \mathrm{tr}(G({\mathbb {I}}_A\otimes P_{jj}^\tau \otimes {\mathbb {I}}_B)G^*)+\mathrm{tr}(G({\mathbb {I}}_A\otimes P_{kk}^\tau \otimes {\mathbb {I}}_B)G^*)-2\mathrm{tr}(({\mathbb {I}}_A\otimes P_{jk}^\tau \otimes {\mathbb {I}}_B)G({\mathbb {I}}_A\otimes P_{kj}^\tau \otimes {\mathbb {I}}_B)G^*). \end{aligned}$$

Spelling out the traces we get

9.11 $$\begin{aligned}&\mathrm{tr}(G({\mathbb {I}}_A\otimes P_{jj}^\tau \otimes {\mathbb {I}}_B)G^*)=\sum _{n,m,r,s}\sum _l \big |\langle \varphi _r^{A}\otimes \varphi _l^\tau \otimes \varphi _s^{B}|G \, \varphi _n^{A}\otimes \varphi _j^\tau \otimes \varphi _m^{B} \rangle \big |^2 \end{aligned}$$

9.12 $$\begin{aligned}&\mathrm{tr}(({\mathbb {I}}_A\otimes P_{jk}^\tau \otimes {\mathbb {I}}_B)G({\mathbb {I}}_A\otimes P_{kj}^\tau \otimes {\mathbb {I}}_B)G^*) \nonumber \\&\quad = \sum _{n,m,r,s} \langle \varphi _r^{A}\otimes \varphi _k^\tau \otimes \varphi _s^{B}|G \, \varphi _n^{A}\otimes \varphi _k^\tau \otimes \varphi _m^{B} \rangle \langle \varphi _n^{A}\otimes \varphi _j^\tau \otimes \varphi _m^{B}|G \, \varphi _r^{A}\otimes \varphi _j^\tau \otimes \varphi _s^{B} \rangle , \end{aligned}$$

and we observe that the complex conjugate of (9.12) is obtained by exchanging *j* and *k*. Hence we can express the real part of (9.10) as

9.13 $$\begin{aligned}&\sum _{n,m,r,s} \big |\langle \varphi _r^{A}\otimes \varphi _j^\tau \otimes \varphi _s^{B}|G \, \varphi _n^{A}\otimes \varphi _j^\tau \otimes \varphi _m^{B} \rangle \big |^2 +\big |\langle \varphi _r^{A}\otimes \varphi _k^\tau \otimes \varphi _s^{B}|G \, \varphi _n^{A}\otimes \varphi _k^\tau \otimes \varphi _m^{B} \rangle \big |^2\nonumber \\&\quad - \langle \varphi _r^{A}\otimes \varphi _k^\tau \otimes \varphi _s^{B}|G \, \varphi _n^{A}\otimes \varphi _k^\tau \otimes \varphi _m^{B} \rangle \langle \varphi _n^{A}\otimes \varphi _j^\tau \otimes \varphi _m^{B}|G \, \varphi _r^{A}\otimes \varphi _j^\tau \otimes \varphi _s^{B} \rangle \nonumber \\&\quad - \langle \varphi _r^{A}\otimes \varphi _j^\tau \otimes \varphi _s^{B}|G \, \varphi _n^{A}\otimes \varphi _j^\tau \otimes \varphi _m^{B} \rangle \langle \varphi _n^{A}\otimes \varphi _k^\tau \otimes \varphi _m^{B}|G \, \varphi _r^{A}\otimes \varphi _k^\tau \otimes \varphi _s^{B} \rangle \nonumber \\&\quad +\big |\langle \varphi _r^{A}\otimes \varphi _k^\tau \otimes \varphi _s^{B}|G \, \varphi _n^{A}\otimes \varphi _j^\tau \otimes \varphi _m^{B} \rangle \big |^2 +\big |\langle \varphi _r^{A}\otimes \varphi _j^\tau \otimes \varphi _s^{B}|G \, \varphi _n^{A}\otimes \varphi _k^\tau \otimes \varphi _m^{B} \rangle \big |^2\nonumber \\&\quad +\sum _{n,m,r,s} \sum _{l\not \in \{j,k\}} \big |\langle \varphi _r^{A}\otimes \varphi _l^\tau \otimes \varphi _s^{B}|G \, \varphi _n^{A}\otimes \varphi _j^\tau \otimes \varphi _m^{B} \rangle \big |^2\nonumber \\&\quad + \big |\langle \varphi _r^{A}\otimes \varphi _l^\tau \otimes \varphi _s^{B}|G \, \varphi _n^{A}\otimes \varphi _k^\tau \otimes \varphi _m^{B} \rangle \big |^2. \end{aligned}$$

With $a= \langle \varphi _r^{A}\otimes \varphi _j^\tau \otimes \varphi _s^{B}|G \, \varphi _n^{A}\otimes \varphi _j^\tau \otimes \varphi _m^{B} \rangle$ and $b=\langle \varphi _r^{A}\otimes \varphi _k^\tau \otimes \varphi _s^{B}|G \, \varphi _n^{A}\otimes \varphi _k^\tau \otimes \varphi _m^{B} \rangle$, we rewrite the first four terms of the summand as

9.14 $$\begin{aligned} |a|^2+|b|^2-b\bar{a} -a\bar{b}=\Big \langle \begin{pmatrix}a \\ b\end{pmatrix}\Big | \begin{pmatrix}1 &{} -1 \\ -1 &{} 1\end{pmatrix}\begin{pmatrix}a \\ b\end{pmatrix}\Big \rangle \ge 0, \end{aligned}$$

since $$\begin{pmatrix}1 &{} -1 \\ -1 &{} 1\end{pmatrix}\ge 0$$. The remaining terms can reorganised as follows,

9.15 $$\begin{aligned}&\sum _{n,m,r,s} \big |\langle \varphi _r^{A}\otimes \varphi _j^\tau \otimes \varphi _s^{B}|G \, \varphi _n^{A}\otimes \varphi _k^\tau \otimes \varphi _m^{B} \rangle \big |^2+\sum _{l\not \in \{j,k\}} \big |\langle \varphi _r^{A}\otimes \varphi _l^\tau \otimes \varphi _s^{B}|G \, \varphi _n^{A}\otimes \varphi _j^\tau \otimes \varphi _m^{B} \rangle \big |^2\nonumber \\&\quad =\sum _{n,m,r,s} \sum _{l\ne j} \big |\langle \varphi _r^{A}\otimes \varphi _l^\tau \otimes \varphi _s^{B}|G \, \varphi _n^{A}\otimes \varphi _j^\tau \otimes \varphi _m^{B} \rangle \big |^2\nonumber \\&\quad = \mathrm{tr}\Big \{({\mathbb {I}}- {\mathbb {I}}_A\otimes P_{jj}^\tau \otimes {\mathbb {I}}_B) G({\mathbb {I}}_A\otimes P_{jj}^\tau \otimes {\mathbb {I}}_B)G^*({\mathbb {I}}-{\mathbb {I}}_A\otimes P_{jj}^\tau \otimes {\mathbb {I}}_B) \Big \} , \end{aligned}$$

and similarly for the terms with second index equal to *k*, which yields the result. $\square$

And the proof of Proposition 6.7 is finished.
